# Natural products in treating sepsis-associated lung and liver injuries by mediating ferroptosis, current progress, and future perspective

**DOI:** 10.3389/fphar.2026.1785916

**Published:** 2026-05-18

**Authors:** Jingjing Li, Xiaolan Xia, Kun Wang, Xia Xu, Chun Li

**Affiliations:** Department of Pharmacy, Wuhou District People’s Hospital, Chengdu, Sichuan Wuhou District Maternal and Child Health Hospital, Chengdu, Sichuan, China

**Keywords:** acute lung injury, ferroptosis, liver injury, natural products, sepsis

## Abstract

Sepsis is a life-threatening disorder triggered by an unregulated host reaction to infection, and it represents a worldwide health dilemma given the scarcity of effective treatment strategies. Sepsis usually leads to lethal multiorgan dysfunctions, including acute liver failure (ALF) and acute lung injury (ALI). Recent studies have found that altered programmed cell death (PCD) processes, including apoptosis, autophagy, ferroptosis, and pyroptosis, belong to key mechanisms that trigger sepsis-associated multiorgan disorders. Among them, ferroptosis is a unique mode of PCD characterized by the accumulation of iron-dependent reactive oxygen species (ROS) and lipid peroxidation. Mediating ferroptosis is a promising strategy against sepsis. Recently, studies have identified multiple natural products for treating sepsis-associated lung and liver injuries by targeting ferroptosis. Here, we provide an overview of the mechanisms and potential therapeutic targets underlying ferroptosis in sepsis. The natural products with ferroptosis targeting will be summarized. Notably, most current evidence supporting the therapeutic potential of natural products is derived from preclinical investigations, and high-quality clinical data remain scarce, requiring further validation for clinical translation. We hope this study provides new perspectives for the future treatment of sepsis-induced ALI and ALF.

## 1 Introduction

Sepsis is a life-threatening organ dysfunction caused by a dysregulated host response to infection (Singer et al., 2016). Sepsis remains a major global health threat, with its disease burden clearly decreasing in children and increasing in adults. Although the number of cases and deaths decreased between 1990 and 2019, influenced by COVID-19, the global burden of sepsis increased in 2021, with an estimated 166 million sepsis cases and 21.4 million sepsis-related deaths worldwide (GBD 2021 Global Sepsis Collaborators, 2025). The lungs are the first and most vulnerable organs affected during sepsis (Zhou and Liao, 2021). Approximately 25%–50% of sepsis patients may develop acute lung injury or even acute respiratory distress syndrome (ARDS) (Sevransky et al., 2009). In sepsis, pathogen-associated molecular pattern (PAMP) stimulation triggers innate immune responses, whereas damage-associated molecular pattern (DAMP) signaling promotes innate immune responses. The excessive release of proinflammatory cytokines (such as TNF-α, IL-1, and IL-6) also leads to systemic coagulation abnormalities, collectively triggering a systemic inflammatory cascade, resulting in immune imbalance and aggravated lung injury. Pulmonary phagocytes and endothelial cells release large amounts of inflammatory factors, activating alveolar macrophages and neutrophils, which generate reactive oxygen species such as superoxide anions. These reactive oxygen species further convert into highly toxic oxidants such as hydrogen peroxide, hydroxyl radicals, and hypochlorous acid, directly damaging the alveolar epithelium and vascular endothelium and exacerbating lung injury (Sun B. et al., 2023). Additionally, approximately one-third of sepsis patients develop sepsis-associated liver injury (SLI), which often predicts poor outcomes (Song Y. et al., 2025). Although the liver has strong regenerative and anti-injury capabilities, it is less prone to damage than other organs. Once SLI occurs, patient mortality even exceeds that caused by lung injury, the most frequently affected organ in sepsis (Sun et al., 2020). The pathophysiological process of SLI is complex and involves mechanisms such as inflammation, oxidative stress, mitochondrial dysfunction, programmed cell death, autophagy, and epigenetic regulation, which are not fully understood and directly lead to clinical treatment challenges (Xu X. et al., 2025).

**FIGURE 1:**
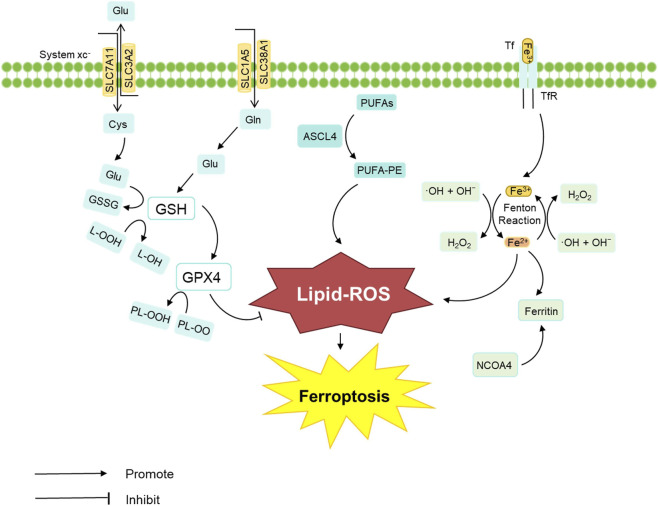
Core regulatory mechanisms of ferroptosis. Ferroptosis is regulated by three major pathways, including the Fenton reaction, System Xc- (SLC7A11), and the GPX4-mediated antioxidant system.

Ferroptosis is a form of regulated cell death (RCD) characterized by excessive iron accumulation and lipid peroxidation (Wang et al., 2023a). Basically, the core pathways of ferroptosis include the Fenton reaction, System Xc- (SLC7A11), and the glutathione peroxidase 4 (GPX4)-mediated antioxidant system (Figure 1). Its morphological features include reduced mitochondrial volume, rupture of the mitochondrial outer membrane, decreased or absent mitochondrial cristae, and a normal-sized nucleus without chromatin condensation, distinguishing it from other modes of death (Stockwell et al., 2017). Ferroptosis plays a significant role in the development of sepsis and its associated organ injuries (such as S-ALI and SLI) (Huo et al., 2023). In ALI, ferroptosis has been shown to damage pulmonary microvascular endothelial cells during sepsis (Shen et al., 2023). Type II alveolar epithelial (AT2) cells are also susceptible to ferroptosis (Dong et al., 2020). As it is the primary iron storage organ in the body, liver injury is closely linked to iron overload. Studies have shown that uncontrolled free iron is hepatotoxic and can contribute to the development of various liver diseases, including viral hepatitis, drug-induced liver injury, and even ALF (Xie et al., 2023).

Natural products are crucial for drug discovery and development and are widely extracted from plants, animals, marine organisms, and microorganisms. The clinical value of various natural product-derived drugs has been confirmed (Liang et al., 2024). Natural products exhibit excellent anti-inflammatory and antioxidant properties and have demonstrated significant therapeutic potential in acute organ injury. For instance, compared with other carotenoids, astaxanthin, a natural carotenoid, possesses superior antioxidant activity and shows notable therapeutic effects in ALI (Alugoju et al., 2023). Panaxadiol, extracted from ginseng roots, alleviates ALI symptoms by inhibiting ferroptosis through upregulation of the Keap1-Nrf2/HO-1 pathway (Li J. et al., 2021). An ethanol extract from Physalis angulata L. fruits can suppress M1 polarization and excessive inflammation in macrophages by modulating PFKFB3 acetylation and phosphorylation, thereby mitigating S-ALI (Liu et al., 2025). Notably, natural products can ameliorate sepsis-induced organ damage by regulating ferroptosis. For example, 5β-hydroxycostic acid isolated from Laggera alata exerts anti-inflammatory and anti-ferroptotic effects by inhibiting the NF-κB and MAPK signaling pathways, thereby alleviating sepsis-associated kidney injury (Li et al., 2025a). Additionally, baicalein, derived from Scutellaria baicalensis roots, inhibits LPS-induced myocardial ferroptosis by regulating the miR-299b-5p/HIF1-α signaling axis, offering myocardial protection in sepsis models (Zhou W. Y. et al., 2025).

Given the significant potential of natural products in the treatment of sepsis-related acute organ injury, we focus on the role and mechanisms through which natural products improve S-ALI and SLI by regulating ferroptosis. This not only provides new ideas and theoretical foundations for the prevention and treatment of organ damage caused by sepsis but also deepens the understanding of how natural products exert therapeutic effects through the regulation of ferroptosis, thereby promoting their translation from basic research to clinical applications.

## 2 Key modulators of ferroptosis in S-ALI and SLI

**FIGURE 2:**
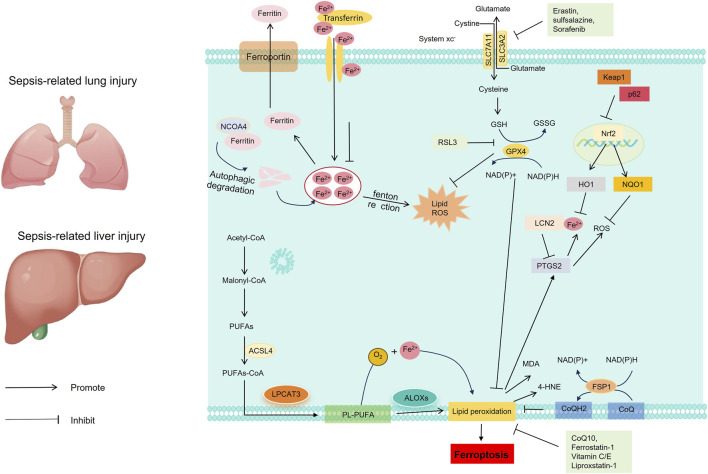
Schematic diagram of the regulatory mechanisms of ferroptosis in S-ALI and SLI. Abbreviations: MDA, malondialdehyde; GSH, reduced glutathione; ROS, reactive oxygen species; 4-HNE, 4-hydroxy-nonenal; NCOA4, nuclear receptor co-activator 4; GPX4, glutathione peroxidase 4; Keap1, Kelch-like ECH-associated protein 1; Nrf2, nuclear factor E2-related factor 2; HO-1, heme oxygenase-1; SLC7A11, solute carrier family 7 member 11; SLC3A2, solute carrier family 3 member 2; ACSL4, acyl-CoA synthase long-chain family member 4; PUFAs, polyunsaturated fatty acids; PUFAs-OOH, polyunsaturated fatty acid hydroperoxides; PTGS2, prostaglandin E2 synthase 2; LCN2: Lipocalin-2.

Iron overload is a key driver of ferroptosis. During sepsis, intracellular Fe^2+^ levels significantly increase, whereas the expression of the iron storage protein FTH1 decreases and the expression of the iron uptake receptor TFR1 increases, collectively exacerbating intracellular iron accumulation (Wang et al., 2018; Dai et al., 2023; Zhang J. et al., 2022). Excess iron generates a large amount of reactive oxygen species (ROS) through the Fenton reaction, creating conditions favorable for ferroptosis (Wang et al., 2018). Ferroptosis plays a critical role in S-ALI and SLI through two core mechanisms: iron homeostasis imbalance and lipid peroxidation (Figure 2). This process is often accompanied by changes in a series of key regulatory molecules of ferroptosis. By modulating abnormal molecular expression, ferroptosis-mediated S-ALI and SLI can be significantly alleviated (Table 1).

**TABLE 1:** Different characteristics of ferroptosis and related targets in S-ALI and SLI.

Categories	S-ALI	SLI
Key cell types	Type II alveolar epithelial cells (AT2), alveolar macrophages, and pulmonary microvascular endothelial cells	hepatocytes, Kupffer cells
Upstream pathways	TLR4/NF-κB, TLR9, Keap1-Nrf2/HO-1, YAP1-TEAD, PERK-ATF4-CHOP	HMGB1/TLR4, IL-6/STAT3, PI3K/AKT/Nrf2
Characteristics of iron metabolism	Mitochondrial iron accumulation and NCOA4-mediated ferritin phagocytosis release of Fe^2+^	Degradation of ferritin, release of free iron, and imbalance of the hepcidin/FPN axis
Alterations of key ferroptosis molecules	SLC7A11 (↓), GPX4 (↓), ACSL4 (↑), YAP1 (↑), METTL3 (↑)	SLC7A11 (↓), GPX4 (↓), ACSL4 (↑), FTH1(↑)
Pathological features	Decreased pulmonary surfactant, alveolar collapse, pulmonary edema, and infiltration of inflammatory cells	Hepatocellular necrosis, liver failure (ALT/AST elevation), and accumulation of lipid peroxidation
Targets of natural products	Hyperoside and Andrographolide (Keap1-Nrf2) Isoginkgetin (TLR4/NF-κB) Puerarin (SLC7A11/GPX4/FTH1) Kaempferol (NSUN7-m5C-TFRC) Ferulic acid (Nrf2/HO-1) Salidroside (ARA metabolism) Curcumin (TXNIP/TRX-1/GPX4) Saikosaponin D (Nrf2) Anemoside B4 (RAGE-Nrf2) XBJ (Nrf2/HO-1)	Nobiletin (Nrf2-Gpx4) Exocarpium Citri Grandis (GPX4/SLC7A11) Wedelolactone (PI3K/AKT/Nrf2) Isoferulic acid (SIRT1) Artemisitene (Nrf2) Coptisine (STAT1/IRF1/GPX4) Tetramethylpyrazine (TFR1/Atf3/GPX4/Nrf2) Irisin (ACSL4/COX-2) Maresin 1(Nrf2/SLC7A11/GPX4)

### 2.1 GPX4

The occurrence of ferroptosis is closely related to the collapse of the cellular antioxidant defense system and the execution of lipid peroxidation. The core mechanisms include reduced activity of GPX4 and depletion of GSH, along with decreased levels of antioxidant enzymes such as SOD and CAT, leading to severely impaired cellular ability to clear lipid peroxides (Jiang et al., 2021; Zeng et al., 2023; Guo Y. et al., 2024; Wei et al., 2020). In this context, polyunsaturated fatty acids (PUFAs) undergo extensive peroxidation catalyzed by enzymes such as arachidonate lipoxygenases (ALOXs), generating toxic products such as malondialdehyde (MDA) and 4-hydroxynonenal (4-HNE) (Gaschler and Stockwell, 2017; Feng and Stockwell, 2018). The accumulation of these lipid peroxidation products disrupts the integrity of cell membranes, ultimately causing cell rupture and ferroptosis (Li X. et al., 2019). Evidence from murine sepsis models has shown that MDA and 4-HNE levels in the lung and liver were significantly increased, confirming the critical role of lipid peroxidation (Zhang et al., 2025a; Zhu et al., 2024).

GPX4 was initially known as PHGPX (phospholipid hydroperoxide glutathione peroxidase) and was first purified from pig liver in 1982 (Ursini et al., 1982). GPX4 is the core molecule involved in the defense against ferroptosis. It converts GSH into oxidized glutathione (GSSG) and reduces toxic lipid peroxides (L-OOH) into nontoxic alcohols (L-OH), thereby effectively preventing the accumulation of lipid peroxides. Once its activity is inhibited, it directly leads to the accumulation of lipid peroxides, triggering ferroptosis (Li et al., 2020). In S-ALI and SLI, the downregulation of GPX4 expression is a common pathological feature (Guo Y. et al., 2024; Lv et al., 2023). Notably, the depletion of GSH directly weakens GPX4 activity, disrupts intracellular redox homeostasis, and subsequently promotes ferroptosis (She et al., 2021). Modulating the gut microbiota can activate the Nrf2 signaling pathway, thereby upregulating GPX4 expression and inhibiting COX2 expression and significantly alleviating ferroptosis and inflammatory responses in SLI (Huang et al., 2023). Inhibiting GPX4 expression in hepatocytes exacerbates mitochondrial damage and lipid peroxidation, further driving ferroptosis and worsening the SLI (Wang et al., 2023b). GPX4 is also crucial for lung protection. Studies by He et al. (He et al., 2022) and Wang et al. (Wang Y. M. et al., 2022) have demonstrated that enhancing GPX4 expression can effectively suppress lipid peroxidation and ferroptosis, thereby exerting therapeutic effects on S-ALI.

### 2.2 SLC7A11

Solute carrier family 7 member 11 (SLC7A11, also known as xCT) and the heavy chain subunit solute carrier family 3 member 2 (SLC3A2) are connected via disulfide bonds to form the Xc^−^ system. This system involves the exchange of intracellular glutamate for extracellular cystine at a 1:1 ratio and is a critical pathway through which cells acquire cysteine (Koppula et al., 2021). The imported cystine is reduced to cysteine (Cys) within the cell, which serves as a key precursor for glutathione (GSH) synthesis (Stockwell et al., 2017). Therefore, the activity of the Xc^−^ system directly affects intracellular GSH synthesis levels, thereby regulating GPX4-mediated anti-ferroptosis activity upstream. The inhibition of SLC7A11, the functional subunit of this system, leads to impaired cystine uptake, reduced GSH synthesis, the subsequent inactivation of GPX4, the accumulation of lipid peroxides, and ultimately ferroptosis (Yuan et al., 2020; Hassannia et al., 2019). Additionally, the Xc^−^ system can be inhibited by small molecules such as erastin and its derivatives, sulfasalazine (SSZ), sorafenib, extracellular glutamate, or its analogs. These interventions effectively induce ferroptosis (Hassannia et al., 2019). In sepsis-associated organ injury, targeting the upregulation of SLC7A11 expression has been shown to be a protective strategy. For example, Wang et al. designed a hydrophobic-force-driving self-assembly based dihydroquercetin (DQ) nanoparticle (DQB@C). In a mouse CLP-induced S-ALI model, DQB@C exerted lung-protective effects by increasing SLC7A11 expression levels and inhibiting ferroptosis (Wang Y. et al., 2024). Neuregulin-4 (Nrg4), an adipokine, has protective effects against metabolic disorders and insulin resistance. In the mouse SLI model, exogenous Nrg4 injection alleviated SLI by increasing hepatic GSH, SLC7A11, and GPX4 levels to suppress ferroptosis while concurrently inhibiting inflammatory responses (Feng et al., 2025). These studies collectively demonstrate that SLC7A11 is a pivotal hub that regulates ferroptosis in sepsis-related organ injury, highlighting its translational value as a therapeutic target.

### 2.3 ACSL4

Acyl-CoA synthetase long-chain family member 4 (ACSL4) is an enzyme that converts fatty acids into fatty acyl-CoA esters and is involved in regulating intracellular lipid synthesis and metabolism. As a key regulator of ferroptosis, the expression level and enzymatic activity of ACSL4 directly determine a cell’s sensitivity to ferroptosis (Wang Y. et al., 2022; Doll et al., 2017). ACSL4 preferentially catalyzes long-chain polyunsaturated fatty acids (PUFAs), such as arachidonic acid (AA) and eicosapentaenoic acid (EPA) (Quan et al., 2021). The overexpression of ACSL4 promotes the catalysis of various PUFAs, alters the cellular lipid composition, and ultimately increases the susceptibility of cells to ferroptosis (Cui et al., 2021). Targeting the inhibition of ACSL4 has become a viable strategy for suppressing ferroptosis. For example, the use of thiazolidinedione to inhibit ACSL4 has been shown to significantly ameliorate ferroptosis-mediated organ damage in mice (Doll et al., 2017). In sepsis-associated organ injury, the regulatory role of ACSL4 in ferroptosis has been confirmed by multiple studies. In the mouse S-ALI model, short-term lactate stimulation upregulated ACSL4 expression, directly inducing lipid peroxidation and mitochondrial-dependent ferroptosis and leading to ROS accumulation and oxidative stress, thereby causing alveolar epithelial cell death (Wu et al., 2024). Carnitine palmitoyltransferase 1A (CPT1A) is a crucial enzyme in fatty acid oxidation that regulates the transport of long-chain fatty acids into the mitochondria for fatty acid β-oxidation. CPT1A can reduce ACSL4 protein expression by promoting its succinylation, thereby inhibiting ferroptosis and alleviating S-ALI (Wang and Sun, 2025). In rats with CLP-induced SLI, deferoxamine treatment significantly reduced ACSL4 protein levels, accompanied by decreased lipid peroxidation, ultimately improving the SLI (Zhang et al., 2025b). These studies collectively indicate that the ACSL4 signaling pathway plays a central role in regulating ferroptosis and is a highly promising intervention target in sepsis-related organ injury.

### 2.4 FSP1

Ferroptosis suppressor protein 1 (FSP1), previously known as apoptosis-inducing factor mitochondrial 2 (AIFM2), is a key inhibitor of ferroptosis (Bersuker et al., 2019). It is recruited to the cell membrane through serine methylation and functions as a redox enzyme (Bersuker et al., 2019). In the process of inhibiting ferroptosis, FSP1 and dihydroorotate dehydrogenase (DHODH) act on the plasma membrane and mitochondrial inner membrane, respectively, to reduce ubiquinone (CoQ) to ubiquinol (CoQH2) (Koppula et al., 2022). CoQH2, as a crucial radical-trapping antioxidant, effectively neutralizes lipid peroxidation radicals, thereby blocking ferroptosis (Koppula et al., 2022). Excessive production and failure to eliminate lipid peroxidation (LPO) are key causes of ferroptosis, with polyunsaturated fatty acid hydroperoxides (PUFA-OOH) being the primary source of LPO. Arachidonic acid (AA) and adrenic acid (AdA) are critical peroxidation substrates. Under the catalysis of ACSL4, LPCAT3, and 15-LOX, these enzymes ultimately form peroxidized phosphatidylethanolamine (PE-AA/AdA-OOH), directly triggering ferroptosis (Li and Li, 2020). Notably, the NADPH/FSP1/CoQ10 axis mediated by FSP1 constitutes a robust antioxidant pathway that is independent of and parallel to the classical GSH/GPX4 axis (Li and Li, 2020). This pathway not only provides cells with dual defenses against lipid peroxidation but also positions FSP1 as a significant potential target for inhibiting ferroptosis. Studies have shown that in a sepsis mouse model, inhibiting Hmox1 effectively activates the FSP1/CoQ10/NADPH pathway, significantly increasing autophagy activity and suppressing ferroptosis in alveolar epithelial cells, thereby alleviating lung injury in mice (Li et al., 2025b). Furthermore, ferroptosis mediated by both GPX4 and FSP1 has been confirmed to participate in the SLI mouse model. Activating ALDH2 and regulating FSP1- and GPX4-mediated ferroptosis can have protective effects on the liver (Li et al., 2023).

### 2.5 PTGS2

Prostaglandin-endoperoxide synthase 2 (PTGS2), also known as COX2, is a key enzyme in the cyclooxygenase (COX) family (Gao et al., 2024). Its expression can be induced by various cell types, such as endothelial cells, epithelial cells, and immune cells, under inflammatory stimuli (Tanaka et al., 2024). It plays a central role in inflammatory responses by catalyzing the conversion of arachidonic acid (AA) into prostaglandins (PGs) (Tanaka et al., 2024). During ferroptosis, PTGS2 has been identified as a critical biomarker and triggering factor. Ferroptosis is accompanied by significant upregulation of PTGS2 activity, which is both a key manifestation of GPX4 deficiency leading to exacerbated lipid peroxidation and an important mechanism through which PTGS2 promotes ferroptosis (Chen S. et al., 2025; Yang et al., 2014). The increase in PTGS2 further modulates the levels of intracellular reactive oxygen species and iron, resulting in the accumulation of lipid peroxides and thereby aggravating iron-dependent cellular damage (Chen S. et al., 2025). Therefore, the upregulation of PTGS2 is considered a suitable marker of lipid peroxidation in ferroptosis (Yang et al., 2014). In organ injury caused by sepsis, the expression and activity of PTGS2/COX2 also play pivotal roles. In the CLP-induced ALI mouse model, COX2 expression was significantly elevated in the lungs of mice (Li Z. et al., 2024). Additionally, studies have shown that alleviating S-ALI is associated with the suppression of overactivation of the TLR4/NF-κB/COX-2 pathway (Zhao et al., 2025). Lipocalin-2 (LCN2), a member of the lipocalin family, is a group of secreted proteins that act as transporters for small lipophilic molecules, including iron and lipopolysaccharides. Research has shown that LCN2 overexpression can mitigate LPS-induced oxidative stress and ferroptosis by inhibiting PTGS2 expression in hepatocytes, thereby exerting a protective effect on the liver (Jiang et al., 2024). These results confirm that PTGS2-mediated oxidative stress and ferroptosis are among the key mechanisms underlying S-ALI and SLI.

## 3 Mechanisms of ferroptosis and targeted strategies

During the progression of S-ALI and SLI, multiple signaling pathways are involved in the regulation of ferroptosis. Modulating these signaling pathways can significantly alleviate sepsis-mediated ferroptosis activation, thereby ameliorating lung and liver injury (Figure 2).

### 3.1 Ferrostatin-1

Ferrostatin-1 (Fer-1) is a compound identified from a small-molecule compound library (Miotto et al., 2020). Initially, Dixon et al. identified Fer-1 as a ferroptosis inhibitor in 2012, demonstrating its ability to inhibit ferroptosis in HT-1080 cells induced by RSL3 or erastin (Dixon et al., 2012). The primary mechanism of action of Fer-1 stems from its aromatic amine structure, which enables it to effectively inhibit lipid peroxidation, thereby blocking the ferroptosis process (Dixon et al., 2012). Additionally, Fer-1 can increase GSH levels to suppress ferroptosis in cells (Skouta et al., 2014) and alleviate ferroptosis caused by excessive activation of p53 (Jiang et al., 2015). In addition to directly inhibiting ferroptosis, Fer-1 also has significant anti-inflammatory effects, reducing the release of proinflammatory cytokines in various organ injury models and exerting corresponding organ-protective effects (Martin-Sanchez et al., 2017; Yamada et al., 2020; Li W. et al., 2019). In sepsis-related injuries, Fer-1 clearly has organ-protective effects. Studies have indicated that Fer-1 can reverse the sepsis-induced decrease in GPX4 expression, inhibit key ferroptosis markers (elevating GSH and SOD and reducing MDA and MPO levels), and lower inflammatory cytokine levels, thereby mitigating lung injury in a mouse sepsis model (Wang Y. M. et al., 2022). In the SLI mouse model, Fer-1 intervention significantly reduces the levels of the oxidative stress products ROS and MDA, increases the activity of the antioxidant enzyme SOD, promotes the recovery of damaged mitochondrial morphology, and markedly alleviates liver damage (Li Y. et al., 2025). In summary, Fer-1 primarily blocks ferroptosis by inhibiting lipid peroxidation but also has anti-inflammatory and antioxidant effects, demonstrating clear protective potential against multiple organ injuries such as S-ALI and SLI.

### 3.2 Regulation of the Keap1-Nrf2 axis

Nrf2, as a leucine zipper nuclear transcription factor, is strongly negatively regulated by Keap1 activity. Under steady-state conditions, the cytoplasmic Keap1 protein binds to Nrf2, inhibiting its activity and maintaining the basal expression of its regulated genes. Under cellular stress, Nrf2 dissociates from Keap1, becomes activated, translocates to the nucleus, and initiates the expression of numerous cytoprotective genes, increasing cell survival capacity (Kensler et al., 2007). Keap1 belongs to the BTB-Kelch protein family (also known as KLHL19), and its functional core lies in the Keap1/Nrf2 dimer complex structure, where two Keap1 molecules bind to one Nrf2 molecule. As an adaptor protein, Keap1 senses intracellular induction signals through its multiple reactive cysteine residues, thereby mediating Nrf2 degradation (Dinkova-Kostova et al., 2017). In iron homeostasis and ferroptosis regulation, Nrf2 maintains the intracellular iron balance by modulating the expression of genes involved in heme synthesis, hemoglobin catabolism, iron storage, and transport, thereby influencing ferroptosis sensitivity (Kerins and Ooi, 2018). Notably, Keap1 inactivation stabilizes Nrf2 and its target genes (such as key components of the SLC7A11/cysteine/glutathione (GSH) axis), promoting resistance to ferroptosis, which is crucial for inhibiting ferroptosis (Cao et al., 2019). Studies have shown that panaxydol alleviates acute lung injury in a sepsis mouse model by upregulating the Keap1-Nrf2/HO-1 pathway and suppressing LPS-induced ferroptosis and inflammation (Li J. et al., 2021). Similarly, ADSC-exos mitigate oxidative stress and endothelial cell ferroptosis by promoting Nrf2 expression and nuclear translocation and upregulating GPX4 expression, ultimately alleviating SLI and reducing mortality in (Wang X. et al., 2025).

### 3.3 Regulation of YAP1 signaling

Yes-associated protein 1 (YAP1), a key effector in the Hippo signaling pathway, plays a central role in regulating organ size, tissue regeneration, and stem cell self-renewal (Zhao et al., 2011). As a transcriptional coactivator, YAP1 is widely involved in regulating cell growth, proliferation, and death (Pavel et al., 2018). Its activity is precisely regulated by the Hippo signaling pathway. When the pathway is activated, it promotes the direct phosphorylation of YAP1/2 kinase, causing its retention in the cytoplasm and degradation. When the pathway is inactivated, unphosphorylated YAP1 translocates to the nucleus and binds to TEAD transcription factors to initiate downstream gene expression (Tang et al., 2025). This regulatory mechanism provides a strategic basis for targeting YAP1 to intervene in cell fate. Recent studies have revealed that YAP1 can influence cellular sensitivity to ferroptosis by regulating ferroptosis-related genes such as SLC7A11 and GPX4 (Wang et al., 2023c). In sepsis-induced liver injury in mice, YAP1 clearly protects against ferroptosis. In SLI, YAP1 inhibits ferroptosis through a dual regulatory mechanism. On the one hand, it upregulates SLC7A11 expression to increase the activity of the antioxidant defense system, including GPX4 and GSH, and suppresses ACSL4 expression to alleviate lipid peroxidation; on the other hand, it inhibits ferritinophagy by disrupting the interaction between NCOA4 and FTH1, reducing Fe^2+^ release and mitochondrial ROS generation and ultimately exerting critical liver protective effects (Wang J. et al., 2022). In a S-ALI mouse model, YAP1 similarly enhances cellular antioxidant capacity by upregulating GPX4 and SLC7A11 expression and inhibiting ACSL4, thereby reducing ROS accumulation and lipid peroxidation (Zhang J. et al., 2022). Concurrently, YAP1 effectively mitigates mitochondrial iron overload, dysfunction, and ROS bursts by suppressing ferritinophagy and downregulating the expression of the mitochondrial iron transporter SFXN1, blocking key steps of ferroptosis and achieving lung protection (Zhang J. et al., 2022).

### 3.4 Regulation of NF-κB signaling

Nuclear factor κB (NF-κB) is a key transcription factor that regulates various physiological and pathological processes, such as inflammation, immunity, cell survival, and death. In the resting state, NF-κB resides in the cytoplasm; upon activation by external stimuli, it translocates into the nucleus to regulate downstream gene expression (Taniguchi and Karin, 2018). The NF-κB family comprises five members, p65 (RelA), RelB, c-Rel, p105/p50, and p100/p52, all of which contain the REL homology domain (RHD) (Yu et al., 2020). Its activation relies primarily on two pathways: the canonical pathway and the noncanonical pathway. The canonical pathway mediates the activation of NF-κB1 p50, RELA, and c-REL (also known as canonical NF-κB family members), which are involved in inflammation, immune responses, cell proliferation, and survival. The noncanonical pathway selectively activates p52 and RELB, which primarily regulate immune cell development (Yu et al., 2020; Sun, 2017). Under inflammatory stimuli (e.g., LPS), the IKK complex is activated, leading to the phosphorylation and degradation of IκB, thereby releasing NF-κB and promoting its nuclear translocation (Liu et al., 2017). Activated NF-κB enhances the expression of various inflammatory genes, including iNOS, COX-2, TNF-α, IL-1β, IL-6, and IL-8 (Tak and Firestein, 2001). This process exacerbates local and systemic inflammatory responses and promotes oxidative stress due to NF-κB-induced ROS accumulation (Zhao C. et al., 2023), which is a key driver of ferroptosis (Zheng D. et al., 2022). Thus, the upregulation of the NF-κB pathway collectively creates an environment prone to ferroptosis. Studies have confirmed that inhibiting the NF-κB pathway can alleviate sepsis progression by directly regulating ferroptosis-related proteins (e.g., upregulating GPX4 and downregulating ACSL4) (Xue et al., 2023) or by reducing systemic inflammation through blocking the IKK/IκBα axis (Oh et al., 2019), providing a rationale for targeting this pathway. Importantly, in a S-ALI rat model, the NF-κB signaling pathway is significantly activated in tissues, as indicated by increased phosphorylation of IKKβ and p65 and increased nuclear translocation of phosphorylated p65. Nuclear-translocated p65 directly promotes the expression of downstream proferroptosis genes (e.g., Cox-2), driving ferroptosis in lung epithelial cells and synergistically promoting M1 macrophage polarization, thereby exacerbating lung injury (Ling et al., 2023). Interventions targeting upstream positive regulators (e.g., Srg3) or directly inhibiting NF-κB activation effectively alleviate S-ALI (Ling et al., 2023). In the SLI mouse model, LPS exacerbates oxidative stress and inflammatory responses by suppressing the cytoprotective Nrf2/HO-1 pathway while activating the proinflammatory NF-κB pathway, collectively inducing liver injury. Studies have demonstrated that inhibiting the NF-κB pathway not only reduces inflammation and oxidative stress but also synergistically enhances the activity of the Nrf2/GPX4 pathway, collectively strengthening resistance to ferroptosis and exerting hepatoprotective effects (Zhao C. et al., 2023).

### 3.5 Regulation of endoplasmic reticulum stress

The endoplasmic reticulum (ER) is a key organelle in eukaryotic cells responsible for storing and regulating calcium release and serves as the entry point for the secretory pathway. Under microenvironmental stimuli, protein misfolding and accumulation occur in the ER, a process termed ER stress (Wang and Kaufman, 2014). To restore ER homeostasis and reduce the number of misfolded or unfolded proteins in the ER, the unfolded protein response (UPR) is activated (Ajoolabady et al., 2023).

The ER stress response (UPR) is initiated and regulated by three ER sensors: inositol-requiring enzyme 1 (IRE1), double-stranded RNA-activated protein kinase R-like ER kinase (PERK), and activating transcription Factor 6 (ATF6) (Chen et al., 2023). Among these pathways, the PERK pathway plays a central role in ERS-induced ferroptosis. For instance, through the PERK-eIF2α-ATF4-CHOP pathway and the PERK-Nrf2-HO-1 pathway, Fe^2+^ accumulation and lipid peroxidation are induced, thereby triggering ferroptosis (Zhao et al., 2021; Wei et al., 2021). In the PERK‒ATF4 pathway, the activation of ATF4 enables the binding of HSPA5 to GPX4, resulting in the formation of a complex that inhibits GPX4 degradation and ferroptosis (Zheng X. et al., 2022). Additionally, PERK can suppress System Xc- via p53, reducing GSH synthesis and ultimately promoting ferroptosis (Zheng X. et al., 2022).

Notably, ferroptosis inducers can also activate the PERK-eIF2α-ATF4-CHOP pathway, inducing the expression of p53-upregulated apoptosis regulator (PUMA) and participating in the ERS process (Lee et al., 2018). They can further inhibit cystine–glutamate exchange in cells, increase the expression of the ERS-related protein CHAC1 (glutathione-specific γ-glutamylcyclotransferase 1), and activate ERS. CHAC1 is involved in the activity of the ATF4-CHOP pathway during ERS (Lee et al., 2018). Studies have shown that lipopolysaccharide-induced ER stress in cells can be downregulated by the ferroptosis inhibitor Fer-1 (Li et al., 2022). In sepsis models, rmMANF exerts antioxidant effects by inhibiting the GRP78/PERK/ATF4 axis, thereby suppressing ERS-induced ferroptosis in tissues and ultimately alleviating S-ALI (Zeng et al., 2023). Similarly, the absence of TMEM16A in AT2 cells can mitigate S-ALI by reducing ERS-related ferroptosis during ALI (Jiang et al., 2023). Although the specific role of ERS in SLI has not been fully elucidated, multiple studies have confirmed that inhibiting ERS-mediated ferroptosis plays a hepatoprotective role in acetaminophen-, alcohol-, and immune-mediated liver injury (Xu et al., 2023; Xu L. et al., 2025; Qian et al., 2025), suggesting that this mechanism may have similar potential in SLI.

### 3.6 Regulation of STAT3 signaling

The signal transducer and activator of transcription (STAT) family was initially discovered as ligand-induced transcription factors and plays a central role in mediating cytokines. This family consists of seven members, STAT1, STAT2, STAT3, STAT4, STAT5a, STAT5b, and STAT6, each of which performs unique functions in immune responses, cell growth, differentiation, and apoptosis (Wong et al., 2022; Dai et al., 2025). As a core member of the STAT family, STAT3 is widely expressed in various mammals. As a crucial transcription factor, STAT3 can be activated under cytokine stimulation, subsequently initiating the transcription of multiple target genes and participating in the regulation of key biological processes such as cell growth, apoptosis, and iron metabolism. Additionally, STAT3 is involved in cell proliferation, angiogenesis, and inflammatory responses and plays significant roles in both physiological and pathological processes. Under normal physiological conditions, STAT3 is localized primarily in the cytoplasm and exists as monomers or nonphosphorylated dimers (Song et al., 2023; Xie et al., 2025; Ouyang et al., 2022). In strategies targeting ferroptosis, the role of STAT3 is particularly critical. By binding to the promoters of key genes such as SLC7A11, GPX4, and FTH1, STAT3 positively regulates cellular resistance to ferroptosis. In line with this, inhibiting STAT3 in gastric cancer cells triggers classical ferroptosis characteristics, including increased lipid ROS levels, Fe^2+^ accumulation, GSH/GSSG depletion, and lipid peroxidation (Ouyang et al., 2022). In non-neoplastic diseases such as sepsis-associated organ injury, the regulation of ferroptosis by STAT3 is context-dependent and may exhibit bidirectional effects. During the S-ALI and SLI processes, STAT3 activity is finely regulated at multiple levels and serves as a potential therapeutic target. For example, TREM2 inhibits STAT3 phosphorylation by activating the tyrosine phosphatase SHP1, thereby alleviating oxidative stress and ferroptosis and significantly mitigating S-ALI (Wu et al., 2025). LPS-treated bovine hepatocytes and iE-DAP-treated mice were used to establish *in vitro* and *in vivo* liver injury models, respectively. Blocking the IL-6/STAT3 pathway inhibited ferritinophagy and suppressed hepcidin/FPN-dependent iron efflux. This reduces abnormal intracellular iron accumulation and the resulting lipid peroxidation, ultimately exerting an inhibitory effect on ferroptosis (Zhang et al., 2025c). In summary, STAT3 is not only a central hub in pathological processes but also a key therapeutic target for alleviating disease progression through multiple pathways (e.g., inhibiting oxidative stress and regulating iron metabolism).

### 3.7 Regulation of the PI3K/AKT/mTOR pathway

The PI3K/AKT/mTOR signaling axis is a critical regulator of key cellular functions, including proliferation, metabolism, survival, and immune regulation (Jiang et al., 2020). Activation of the PI3K/AKT/mTOR pathway begins when growth factors or cytokines activate PI3K through receptor tyrosine kinases (RTKs) or G protein-coupled receptors (GPCRs), which then catalyze the conversion of PIP2 to PIP3, recruiting and activating AKT (Glaviano et al., 2023). AKT promotes cell survival by phosphorylating and inhibiting pro-apoptotic proteins (such as BAD and FOXO) and activating pro-survival proteins (such as MDM2) (Glaviano et al., 2023). Additionally, AKT phosphorylates and inhibits the TSC complex, causing TSC to dissociate from the lysosomal surface and subsequently activating Rheb. The GTP-bound form of Rheb directly binds to the catalytic domain of mTOR, thereby activating mTORC1 (Popova and Jücker, 2021). mTORC1 promotes protein synthesis and cell growth by phosphorylating S6K1 and 4E-BP1, whereas mTORC2 further phosphorylates AKT, resulting in the formation of a positive regulatory loop (Popova and Jücker, 2021).

This pathway is also closely associated with ferroptosis. Studies have shown that downregulating the PI3K/AKT/mTOR pathway can increase cellular sensitivity to ferroptosis by downregulating the expression of downstream factors such as GPX4, SREBP1, and SLC7A11 (Jiang et al., 2020). As a critical regulatory axis of ferroptosis, this pathway has potential value in sepsis injury protection. For example, the microbial metabolite 3-hydroxybutyrate (3-HB) can activate the PI3K/AKT/mTOR pathway, upregulate the key lipid metabolism factor LPIN1, and inhibit hepatocyte ferroptosis, manifested by reduced levels of ACSL4, MDA, lipid peroxides (LPO), and Fe^2+^, ultimately alleviating SLI in mice (Hu et al., 2025). Although there is currently a lack of literature directly elucidating the regulation of ferroptosis by the PI3K/AKT/mTOR pathway in S-ALI, existing studies have confirmed its key role in inflammation regulation, apoptosis, and barrier protection in S-ALI/ARDS. For instance, in a rat model with S-ALI, activation of the PI3K/AKT/mTOR pathway can upregulate VCAM-1 and ICAM-1 expression, promoting neutrophil-endothelial cell adhesion and thereby triggering and amplifying uncontrolled inflammatory cascades. Moreover, this pathway can increase the expression of the proapoptotic protein Bax and inhibit the antiapoptotic protein Bcl-2, leading to apoptosis imbalance and further disruption of pulmonary barrier function (Zhou et al., 2024). Therefore, the PI3K/AKT/mTOR pathway may exert organ-protective effects in S-ALI by regulating ferroptosis processes. These findings suggest that the PI3K/AKT/mTOR pathway exerts organ-specific regulatory effects on ferroptosis and the progression of sepsis. In the liver, its activation enhances antioxidant and anti-ferroptotic defense mechanisms. In the lung, excessive activation promotes inflammatory infiltration and barrier disruption. These divergent outcomes may arise from distinct downstream transcriptional programs and cell-type-specific effector molecules, which remain to be further elucidated.

### 3.8 Targeting lncRNAs and miRNAs

**FIGURE 3:**
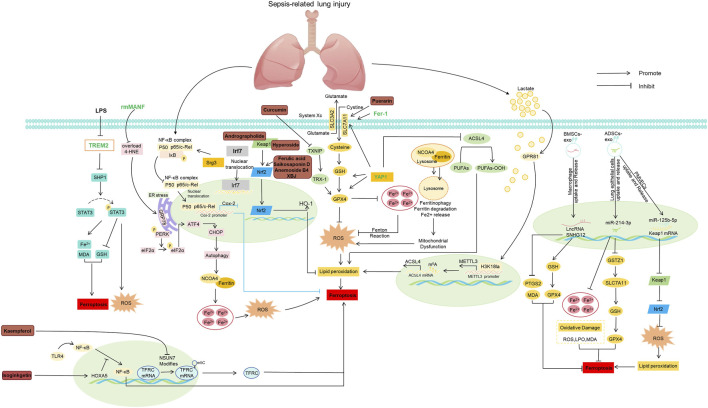
Molecular mechanisms of natural products for treating sepsis-induced acute lung injury by regulating ferroptosis. Abbreviations: 4-HNE, 4-Hydroxynonenal; ACSL4, Acyl-CoA Synthetase Long-Chain Family Member 4; ADSCs, Adipose-Derived Stem Cells; ATF4, Activating Transcription Factor 4; BMSCs, Bone Marrow Mesenchymal Stem Cells; CHOP, C/EBP Homologous Protein; Cox-2, Cyclooxygenase-2; eIF2α, Eukaryotic Translation Initiation Factor 2α; ER stress, Endoplasmic Reticulum Stress; Fer-1, Ferrostatin-1; GPR81, G Protein-Coupled Receptor 81; GPX4, Glutathione Peroxidase 4; GRP78, Glucose-Regulated Protein 78; GSH, Glutathione; GSTZ1, Glutathione S-Transferase Zeta 1; H3K18la, Histone H3 Lysine 18 Lactylation; HO-1, Heme Oxygenase-1; HOXA5, Homeobox A5; IkB, Inhibitor of Nuclear Factor Kappa B; Irf7, Interferon Regulatory Factor 7; Keap1, Kelch-Like ECH-Associated Protein 1; LncRNA SNHG12, Long Non-Coding RNA Small Nucleolar RNA Host Gene 12; LPO, Lipid Peroxidation; LPS, Lipopolysaccharide; m^5^C, 5-Methylcytosine; m^6^A, N6-Methyladenosine; MDA, Malondialdehyde; METTL3, Methyltransferase Like 3; miR-125b-5p, MicroRNA-125b-5p; miR-214-3p, MicroRNA-214-3p; NCOA4, Nuclear Receptor Coactivator 4; NF-κB, Nuclear Factor Kappa B; Nrf2, Nuclear Factor Erythroid 2-Related Factor 2; NSUN7, NOP2/Sun RNA Methyltransferase Family Member 7; PERK, Protein Kinase R-Like Endoplasmic Reticulum Kinase; PMECs, Pulmonary Microvascular Endothelial Cells; PTGS2, Prostaglandin-Endoperoxide Synthase 2; PUFAs, Polyunsaturated Fatty Acids; PUFAs-OOH, Polyunsaturated Fatty Acid Hydroperoxides; rmMANF, Recombinant Human Mesencephalic Astrocyte-Derived Neurotrophic Factor; ROS, Reactive Oxygen Species; SHP1, Src Homology 2 Domain-Containing Phosphatase 1; SLC3A2, Solute Carrier Family 3 Member 2; SLC7A11, Solute Carrier Family 7 Member 11; Srg3, Serine/Arginine-Rich Splicing Factor 3; STAT3, Signal Transducer and Activator of Transcription 3; System Xc^−^, Cystine/Glutamate Antiporter System Xc^−^; TFR C, Transferrin Receptor; TLR4, Toll-Like Receptor 4; TNIP, TNFAIP3 Interacting Protein; TREM2, Triggering Receptor Expressed on Myeloid Cells 2; TRX-1, Thioredoxin-1; TXNIP, Thioredoxin-Interacting Protein; YAP1, Yes-Associated Protein 1.

In the human genome, more than 90% of RNAs are noncoding RNAs (ncRNAs). Among these, those with a length of less than 50 nucleotides (nt) are classified as small noncoding RNAs, such as microRNAs (miRNAs), while those longer than 200 nucleotides are termed long noncoding RNAs (lncRNAs) (Yue et al., 2023). Studies have confirmed that ncRNAs can regulate ferroptosis-related pathways by targeting core regulatory factors of ferroptosis (e.g., GPX4, SLC7A11, and ACSL4) (Alimohammadi et al., 2025). Specifically, miRNAs can downregulate SLC7A11, enhancing cellular sensitivity to ferroptosis, whereas lncRNAs can stabilize or inhibit pathways that prevent lipid peroxidation (Alimohammadi et al., 2025). Accumulating evidence suggests that modulating miRNAs and lncRNAs can influence the pathological progression of sepsis-related organ damage via ferroptosis pathways. In mice with LPS-induced lung injury, multiple studies have revealed the protective role of ncRNAs in inhibiting ferroptosis and alleviating tissue damage. The lncRNA SNHG12 in bone marrow mesenchymal stem cell-derived exosomes (BMSC-exos) increases the levels of GSH and GPX4 while reducing the levels of MDA and PTGS2, thereby suppressing macrophage ferroptosis, mitigating LPS-mediated MLE-12 cell injury and CLP-induced lung injury in mice, and increasing survival rates of septic mice (Deng et al., 2025). miR-214-3p in adipose-derived stem cell exosomes (ADSCs-exos) attenuates ferroptosis in lung epithelial cells by targeting GSTZ1, ameliorating lung injury (Li M. et al., 2025). Additionally, miR-125b-5p in ADSC-exos alleviated inflammation-induced ferroptosis in pulmonary microvascular endothelial cells by regulating the Keap1/Nrf2/GPX4 axis (Shen et al., 2023). In liver injury, the lncRNA SNGH11 has been shown to mediate ferroptosis in SLI cells via the miR-324-3p/GPX4 axis (Yang et al., 2023). Notably, certain ncRNAs promoted ferroptosis under specific conditions, exacerbating injury. For example, miR-223-3p in LPS-stimulated extracellular vesicles (LPS-EVs) enhances Hippo signaling by targeting MEF2C, thereby promoting autophagy and ferroptosis in alveolar macrophages and aggravating S-ALI (Ye et al., 2025). In summary, through diverse molecular mechanisms, miRNAs and lncRNAs constitute critical components of the ferroptosis regulatory network. In sepsis-induced organ injury, they not only exhibit tissue specificity and functional heterogeneity but also provide potential targets and intervention strategies for ncRNA-based therapies, such as the delivery of protective ncRNAs via exosomes or the use of antagonists to inhibit pathogenic ncRNAs (Ma et al., 2025) (Figure 3)

### 3.9 METTL3-mediated N6-methyladenosine

N6-methyladenosine (m6A) is the most prevalent and conserved type of internal RNA modification, extensively regulating the functions of various RNAs, such as messenger RNA and long noncoding RNA, at the posttranscriptional level and participating in numerous biological processes (Li Y. et al., 2021; Shen et al., 2022). m6A modification is mediated by methyltransferases, with methyltransferase-like 3 (METTL3) being the key catalytic subunit (Shen et al., 2022). In regulating ferroptosis, METTL3 determines cellular sensitivity to ferroptosis by influencing the m6A modification levels of key genes. Studies conducted in hepatoblastoma cells have shown that METTL3 can synergize with the recognition protein IGF2BP1 to increase the stability of SLC7A11 mRNA in a m6A-dependent manner, thereby upregulating its expression and increasing cellular resistance to ferroptosis (Liu et al., 2022). The applicability of this mechanism to normal hepatocytes in sepsis-associated liver injury. However, it requires further validation.

METTL3 also serves as a critical negative regulator of GPX4 expression; its overexpression significantly reduces GPX4 protein levels, exacerbating lipid peroxidation and ferroptosis, and targeted inhibition of METTL3 can affect the m6A modification level of GPX4, ultimately affecting cellular sensitivity to ferroptosis (Meng et al., 2024). Additionally, METTL3 promotes ACSL4 protein expression by increasing ACSL4 mRNA stability through the m6A reader protein YTHDC1, thereby driving ferroptosis (Wu et al., 2024). In S-ALI, neutrophil extracellular traps (NETs) upregulate METTL3 expression by activating the TLR9/MyD88/NF-κB signaling pathway (Zhang H. et al., 2022). High METTL3 expression acts as a key negative regulator of GPX4, mediating m6A modifications on its mRNA to promote GPX4 degradation and induce ferroptosis in alveolar epithelial cells (Wang H. et al., 2024). On the other hand, it can also exacerbate ferroptosis by modifying HIF-1α in a m6A-IGF2BP2-dependent manner, while inhibiting NET formation can alleviate such damage (Zhang et al., 2023). Furthermore, METTL3/YTHDC1-mediated increases in ACSL4 m6A modification and stability promote ferroptosis and tissue damage in S-ALI (Wu et al., 2024). Although direct evidence is lacking, studies indicate significant m6A reprogramming of specific genes in SLI models, with these genes enriched in ferroptosis-related pathways such as lipid metabolism and glutathione metabolism (Li L. et al., 2025).

**FIGURE 4:**
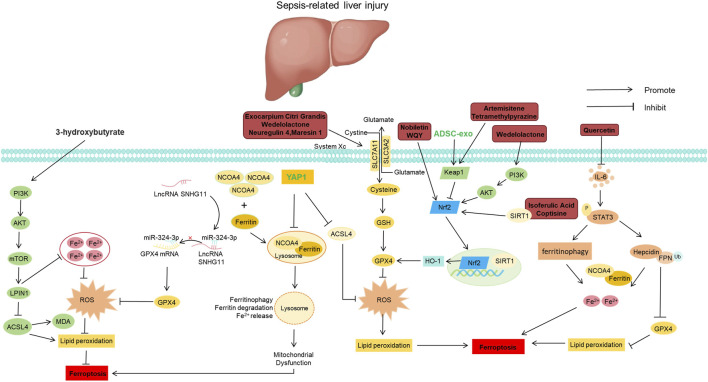
Molecular mechanisms of natural products for treating sepsis-induced liver injury by regulating ferroptosis. Abbreviations: ACSL4, Acyl-CoA Synthetase Long-Chain Family Member 4; ADSC-exo, Adipose-Derived Stem Cell Exosome; AKT, Protein Kinase B; FPN, Ferroportin; GPX4, Glutathione Peroxidase 4; GSH, Glutathione; HO-1, Heme Oxygenase-1; IL-6, Interleukin-6; Keap1, Kelch-Like ECH-Associated Protein 1; LncRNA SNHG11, Long Non-Coding RNA Small Nucleolar RNA Host Gene 11; LPIN1, Lipin 1; MDA, Malondialdehyde; miR-324-3p, MicroRNA-324-3p; mTOR, Mammalian Target of Rapamycin; NCOA4, Nuclear Receptor Coactivator 4; Nrf2, Nuclear Factor Erythroid 2-Related Factor 2; PI3K, Phosphoinositide 3-Kinase; ROS, Reactive Oxygen Species; SIRT1, Sirtuin 1; SLC3A2, Solute Carrier Family 3 Member 2; SLC7A11, Solute Carrier Family 7 Member 11; STAT3, Signal Transducer and Activator of Transcription 3; System Xc^−^, Cystine/Glutamate Antiporter System Xc^−^; Ub, Ubiquitin; YAP1, Yes-Associated Protein 1.

Given that METTL3 bidirectionally regulates key ferroptosis genes through m6A (Wu et al., 2024; Liu et al., 2022; Meng et al., 2024), we hypothesize that in SLI, modulating METTL3 may alter the expression of ferroptosis-related factors by influencing the m6A modification levels of genes involved in lipid metabolism and antioxidant defense, thereby regulating hepatocyte ferroptosis and contributing to disease progression (Figure 4). These findings indicate that the regulation of ferroptosis by METTL3 is markedly in a context-dependent manner. This bidirectional modulation may arise from the following factors. First, the functional outcome of METTL3 depends on the type of m6A reader protein it recruits—binding with IGF2BP1 tends to stabilize SLC7A11 and inhibit ferroptosis (Liu et al., 2022), whereas interaction with YTHDC1 promotes ACSL4 expression and induces ferroptosis (Wu et al., 2024). Second, although METTL3 negatively regulates GPX4 and positively regulates SLC7A11, which appears contradictory (Liu et al., 2022; Wang H. et al., 2024), this may reflect differences in METTL3 target gene selectivity across distinct cell types or under varying stress conditions. Therefore, before targeting METTL3 therapeutically, its predominant role in specific organs, cell types, and stages of sepsis must be clearly defined.

### 3.10 Other mediators

In addition to the aforementioned mechanisms, various other mediators and pathways are involved in the occurrence and development of S-ALI and SLI through the regulation of ferroptosis. For example, the inhibition of pentraxin-3 (Ptx3) can alleviate SLI through mechanisms involving the suppression of hepatocyte ferroptosis, a reduction in CCL20/CCR6 axis-mediated M1 macrophage recruitment, and the blockade of the NF-κB pathway-induced polarization of M0 macrophages to the M1 phenotype (Wang H. et al., 2024). GBP2 in macrophage-derived extracellular vesicles interacts with vascular endothelial cell OTUD5, promoting GPX4 ubiquitination and degradation, increasing cellular ferroptosis, and exacerbating S-ALI (Li Z. et al., 2025). Activation of the ALX/PKA/CREB signaling pathway can upregulate GPX4 expression, thereby inhibiting LPS-induced ferroptosis and providing a potential therapeutic strategy for S-ALI in mice (Lv et al., 2023). Serum Pyk2 levels are negatively correlated with iron levels, and targeted inhibition of Pyk2 can alleviate S-ALI in mice, reduce inflammatory responses, and regulate the expression of ferroptosis-related proteins (such as GPX4 and ACSL4) (Wang et al., 2023d). Sivelestat significantly reduces ferroptosis severity and improves survival rates of a mouse S-ALI model by inhibiting NET formation, protecting pulmonary vascular endothelial glycocalyx (SDC-1/HS) integrity, and activating the HGF/cMET pathway (Fei et al., 2024).

## 4 Targeting ferroptosis with natural products for the treatment of S-ALI and SLI

**FIGURE 5:**
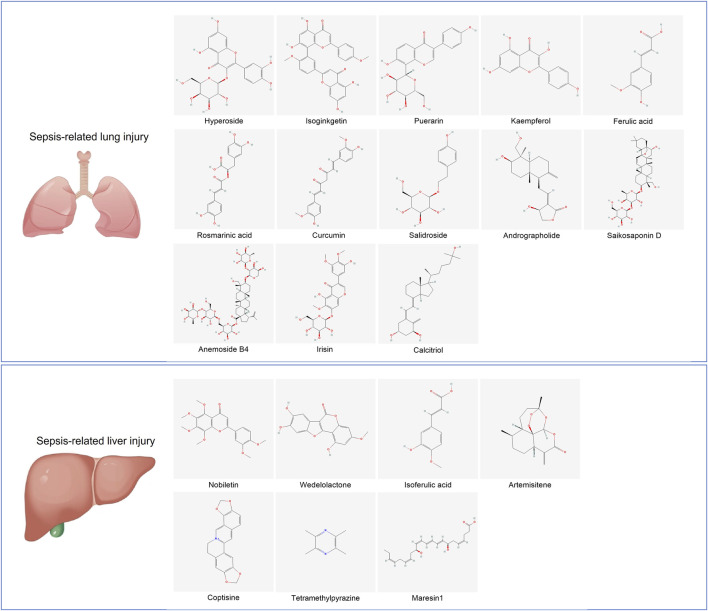
Molecular structure diagram of small-molecule active substances targeting ferroptosis.

Here, we summarize the regulatory effects of natural products on ferroptosis in S-ALI and SLI (Tables 2, 3). The molecular structures of bioactive small molecules derived from natural products are presented in Figure 5.

**TABLE 2:** Natural products improve S-ALI by modulating ferroptosis.

Name	Source	Models	Mechanism	Functions	References
Hyperoside	Flavonoid glycosides found in fruits, vegetables, flowers, and medicinal plants	CLP	Activate the Nrf2 pathway	GPX4 and SLC7A11 can be activated	Chen K. et al. (2025)
Isoginkgetin	Flavonoids extracted from Ginkgo biloba	LPS	Inhibit the TLR4/NF-κB signaling pathway	Reduce the production of ROS, MDA, and Fe2+, and increase the level of GSH	Lu et al. (2025)
Puerarin	Flavonoid monomer	LPS	Inhibit ferroptosis	Reduce lipid peroxidation, decrease ROS production, activate SLC7A11, GPX4, and FTH1, and lower total iron levels and ferrous ion levels	Xu et al. (2021)
Kaempferol	Flavonoid	LPS	Inhibite the NSUN7-mediated TFRCm5C methylation	Reduce the production of ROS, MDA, and Fe2+, and increase the level of GSH	Zhang Y. et al. (2025)
Ferulic acid	Plants and vegetables	CLP/LPS	Activate the Nrf2/HO-1 pathway	Reduce the production of ROS and MDA, and increase the levels of GSH and GFX4	Tang et al. (2022)
Rosmarinic acid	Polyphenolic secondary metabolites derived from rosemary	LPS	Restore the balance of ACE/ACE2 in the renin-angiotensin system	Increase the levels of GSH and GPX4, and decrease the concentrations of Fe2+ and MDA.	Zeng et al. (2024)
Curcumin	Rhizome of Curcuma	CLP/LPS	Inhibit TXNIP and activate the TRX-1/GPX4 pathway	Reduce the production of inflammatory cytokines (IL-1β, IL-6, TNF-α) and lower the levels of oxidative stress markers (ROS, MDA, MPO) as well as Fe2+ content	Chen et al. (2024)
Salidroside	Tocinol glycosides extracted from Rhodiola rosea	CLP	Supress ferroptosis	Reduce the production of ROS, MDA, and Fe^2+^, and increase the level of GSH	Zhen et al. (2024)
Andrographolide	Bioactive compounds extracted from Andrographis paniculata	LPS	Activate the Keap1/Nrf2 pathway	Alleviate LPS-induced pulmonary edema in ALI mice, reduce inflammatory cell infiltration, and mitigate ferroptosis in lung tissue	Li Y. et al. (2024)
Saikosaponin D	Bupleurum chinensis	CLP	Activate the Nrf2/HO-1 pathway	Improved the alveolar epithelial barrier function in CLP-induced septic mice and inhibited ferroptosis	Song Y. et al. (2025)
Anemoside B4	Bioactive saponins isolated from the roots of Pulsatilla chinensis	CLP/LPS	Restrain the AGE/RAGE-Nrf2 axis	Upregulation of GPX4 and SLC7A11 to inhibit macrophage ferroptosis, synergistically exerting anti-inflammatory (reducing IL-1β, TNF-α, IL-6) and antioxidant (reducing MDA, DHE, and increasing GSH) effects	Bu et al. (2025)
Irisin	Skeletal muscle and adipose tissues	CLP	Inhibite ferroptosis and lipid peroxidation with inflammation	Reducing pro-inflammatory factors, increasing GSH and GFX4 levels, and decreasing ROS, MDA, and Fe^2+^ production, along with downregulating the expression of ACSL4, COX-2, and p-AMPK, collectively alleviate oxidative stress and regulate ferroptosis-related proteins	Zhang Y. et al. (2025)
QiShengYiQi pill	Chinese medicinal formulation	CLP	Activate anti-inflammatory and antioxidant pathways	Inhibition of COX-2 and RAGE reduces inflammation. Elevated levels of GSH, GPX4, and SLC7A11 decrease the production of ROS and MDA, thereby increasing the GSH/GSSG ratio	Li Z. et al. (2024)
Xuebijing	Chinese drugs pharmaceutics	LPS	Activate of the Nrf2/HO-1 axis and inhibit GPX4	Induce ferroptosis in CCR2^hi^ monocytes, thereby reducing the infiltration of this pro-inflammatory subset in the lungs, lowering local levels of pro-inflammatory cytokines, and inhibiting the polarization of pulmonary macrophages to the M1 phenotype	Yang et al. (2025)
Calcitriol	The active hormone form produced by vitamin D through two-step enzymatic hydroxylation reactions in the human liver and kidneys	CLP	Correct the dysregulated Ang/Tie2 signaling pathway; regulate the homeostasis of metal ions in the lungs, inhibit ferroptosis and copper death; alleviate systemic and pulmonary inflammation	Increases tight junction protein levels, improves the integrity of the alveolar-capillary barrier, alleviates pulmonary edema and tissue damage; reduces Fe^2+^ levels in the lungs and related proteins (TFRC, ACSL4), upregulates the antioxidant system (FSP-1, SLC7A11, Nrf2, GSH, GPX4), thereby reducing lipid peroxidation and ferroptosis; and attenuates the excessive inflammatory response induced by sepsis	Powała et al. (2024)

**TABLE 3:** Natural products improve SLI by modulating ferroptosis.

Name	Source	Models	Mechanism	Functions	References
Nobiletin	Plant-based polymethoxyflavone	CLP	Upregulate Nrf2/Gpx4 aix	Upregulation of Gpx4 and downregulation of COX2 levels, reduction of iron levels, lipid peroxidation, and ROS production	Huang et al. (2023)
Exocarpium Citri Grandis	Exocarpium Citri Grandis	LPS	Inhibit ferroptosis	Reduced iron content and FTH levels, while increasing the expression of SOD2, SLC7A11, and GPX4	Zhang F. et al. (2025)
Wedelolactone	Coumarin compound derived from Eclipta prostrata	CLP	Activate the PI3K/AKT/NRF2 and SLC7A11/GPX4 signaling pathways	Reduce ROS production and concomitantly upregulate the expression of HO-1, GPX4, and SLC7A11	Yin et al. (2025)
Isoferulic acid	Phenolic compounds derived from the rhizome tissue of Arisaema plants	CLP	Activate the SIRT1–Nrf2 pathway	Significantly enhanced GPX4 expression, GSH levels, and SOD activity, while inhibiting the accumulation of MDAROS and reactive iron ions	Gan et al. (2026)
Artemisitene	Sweet wormwood herb	LPS	Activate Nrf2/HO-1 and inhibition of the NF-κB pathway	The elevation of GPX4, SOD, GSH, and HO-1 levels enhanced cellular antioxidant defense capacity, while simultaneously reducing MDA and ROS levels and alleviating inflammatory responses	Zhao Y. et al. (2023)
Coptisine	Natural alkaloid	CLP/LPS	Activate the STAT1/IRF1/GPX4 pathway	Enhance expression of GSH, GSH/GSSG ratio, SLC7A11, and GPX4, accompanied by reduced production of reactive iron, MDA, and ROS.	Zhu X. et al. (2025)
Tetramethylpyrazine	Chinese herbal extract	CLP/LPS	Inhibite ferroptosis	Inhibit the TFR1 and Atf3, while enhancing the expression of GPX4, Nrf2, and FSP1	Wang J. et al. (2025)
Neuregulin 4	Brown adipose tissue	CLP	Activate the GSH/GPX4 aix	Significantly increase the expression of GSH, SLC7A11, and GPX4, while simultaneously downregulating the levels of Fe^2+^, MDA, and ferritin	Feng et al. (2025)
Maresin1	Endogenous polyunsaturated fatty acid-derived lipids that promote separation	CLP	Activate the Nrf2/SLC7A11/GPX4 pathway	Elevate GSH levels and the GSH/GSSG ratio effectively reduced iron ion and MDA levels	Guo Y. et al. (2024)
Wenqingyin	Chinese herbal extract	LPS	Activate the Nrf2 pathway	Enhance the expression of GSH, SLC7A11, GPX4, SOD, HO-1, and FSP1, while reducing the production of MDA and ROS.	Xie et al. (2023)

### 4.1 Flavonoids

In recent years, various flavonoids have been shown to regulate key pathways involved in ferroptosis. Quercetin, a natural flavonoid, inhibits ferroptosis by reducing harmful MDA and lipid ROS levels while increasing protective GSH levels, thereby alleviating acute kidney injury (Wang et al., 2020). In sepsis-related organ injuries, flavonoids also demonstrate protective potential. For example, hyperoside is a flavonoid glycoside widely present in fruits, vegetables, flowers, and medicinal plants. It preferentially binds to arginine 380 and arginine 415 of the Keap1 protein, thereby inhibiting the ubiquitination and degradation of Nrf2, promoting the translocation of Nrf2 into the nucleus, and upregulating the expression of antiferroptosis-related genes. Studies have indicated that hyperoside suppresses ferroptosis by activating the Nrf2 signaling pathway, resulting in significant protective effects against S-ALI in mice (Chen K. et al., 2025).

Isoginkgetin, a natural bioflavonoid with antioxidant and immunomodulatory activities, has been shown to alleviate LPS-induced ferroptosis in pulmonary epithelial cells and improve the M1/M2 polarization imbalance in LPS-stimulated macrophages via HOXA5-mediated inhibition of the TLR4/NF-κβ signaling pathway, thereby exerting protective effects in S-ALI in BEAS-2B cells (Lu et al., 2025).

Puerarin, a flavonoid monomer, has been demonstrated to mitigate LPS-induced A549 cell injury and downregulate the expression of the inflammatory cytokines TNF-α, IL-8, and IL-1β. In LPS-treated A549 cells, puerarin significantly reduced total iron and Fe^2+^ levels, decreased the MDA content and ROS levels, but increased the GSH level. Additionally, puerarin upregulated the expression of the ferroptosis-related proteins SLC7A11, GPX4, and FTH1 and suppressed NOX1 expression. These findings suggest that puerarin alleviates S-ALI by inhibiting ferroptosis (Xu et al., 2021).

Kaempferol, a flavonoid compound, attenuates ferroptosis in MLE-12 pulmonary epithelial cells during S-ALI by inhibiting NSUN7-mediated m5C methylation of mitochondrial iron transporter protein (TFRC) mRNA (Zhang Y. et al., 2025).

Nobiletin, a plant-derived polymethoxyflavonoid, modulates the gut microbiota in an SLI mouse model, activates the Nrf2 signaling pathway and its downstream HO-1 expression, and further upregulates the Nrf2-Gpx4 signaling axis, thereby inhibiting ferroptosis and exerting hepatoprotective effects (Huang et al., 2023).

Exocarpium Citri Grandis, which is derived from pomelo peel, is rich in flavonoids (such as naringin and neohesperidin) and coumarins. It inhibits ferroptosis through GPX4/SLC7A11 upregulation and normalization of iron overload, synergistically regulating inflammatory and antiapoptotic pathways to alleviate SLI effectively in male C57BL/6J mice (Zhang G. et al., 2025).

### 4.2 Phenolic acids and polyphenols

In recent years, natural phenolic acids and polyphenolic compounds have garnered widespread attention because of their ability to regulate ferroptosis, offering potential for the development of new therapeutic strategies (Zhang and Xie, 2024). Phenolic acids and polyphenolic compounds are a class of natural products widely present in plants that exhibit significant antioxidant and anti-inflammatory activities. Studies have shown that these compounds can exert important protective effects on S-ALI and SLI by modulating key ferroptosis pathways.

Wedelolactone is a coumarin-type polyphenol extracted from Eclipta prostrata (Wang X. Q. et al., 2022). It alleviates SLI in C57BL/6 mice and reduces serum ALT, AST, ROS, and inflammatory cytokine levels, with its protective effects primarily achieved through inhibition of ferroptosis and oxidative stress via the PI3K/AKT/Nrf2 and SLC7A11/GPX4 signaling pathways (Yin et al., 2025).

Isoferulic acid is a hydroxycinnamic acid-type phenolic acid widely found in plant leaves, fruits, and seeds (Singh Tuli et al., 2022). Isoferulic acid (IFA) is a natural phenolic acid present in Cimicifuga plants. Research has indicated that IFA inhibits hepatocyte ferroptosis by regulating the SIRT1 pathway, thereby exerting protective effects against SLI in mice (Gan et al., 2026).

Ferulic acid is a phenolic compound derived from plants and vegetables. In female BALB/c mice with S-ALI, rerulic acid activates the Nrf2/HO-1 pathway to suppress ferroptosis in alveolar epithelial cells, significantly alleviating lung damage, reducing the lung wet/dry weight ratio and myeloperoxidase activity, and improving alveolar epithelial barrier function (Tang et al., 2022). Rosmarinic acid is the main polyphenolic component in rosemary. In a LPS-induced ARDS mouse model, rosmarinic acid inhibits neutrophil and monocyte infiltration in the lungs, increases GPX4 and ACE2 protein expression, improves pulmonary function, and reduces inflammatory cytokine levels, thereby mitigating sepsis-associated ARDS (Zeng et al., 2024). Curcumin is a polyphenolic compound derived from turmeric rhizomes. It inhibits ferroptosis and alleviates S-ALI in mice by increasing TRX-1 and GPX4 levels while reducing TXNIP levels in the lung (Chen et al., 2024). Salidroside is a phenolic glycoside extracted from Rhodiola. In S-ALI, salidroside significantly improved lung function and reduced pathological damage. Its protective mechanism is closely related to the inhibition of ferroptosis and modulation of arachidonic acid metabolism, suggesting that salidroside may mediate the arachidonic acid metabolic pathway to suppress ferroptosis and alleviate S-ALI in mice (Qu et al., 2022; Zhen et al., 2024).

### 4.3 Terpenoids

In recent years, terpenoids have gained widespread attention as important regulators of ferroptosis. For example, dihydroartemisinin, parthenolide, oridonin, triptolide, and oleanolic acid have been confirmed to affect the ferroptosis process through different pathways (Guo J. et al., 2024). Notably, in sepsis-related organ injury models, various terpenoid components have been demonstrated to have protective effects through the targeting of ferroptosis. For instance, andrographolide is a diterpene lactone compound extracted from Paniculata (Islam et al., 2018). Studies have shown that in an LPS-induced ALI mouse model, andrographolide can target the TLR4 receptor, exert significant anti-inflammatory effects by modulating the Keap1/Nrf2 pathway, and effectively inhibit ferroptosis, thereby alleviating lung damage (Li Y. et al., 2024). Saikosaponin D, a triterpenoid saponin extracted from Bupleurum, can maintain the integrity of the alveolar epithelial barrier and improve S-ALI in mice by activating the Nrf2 signaling pathway to inhibit ferroptosis (Song L. et al., 2025). Artemisitene, a natural sesquiterpene compound extracted from *Artemisia annua* (Zhu L. et al., 2025), has been proven to alleviate hepatic oxidative stress and inflammatory responses by inhibiting NF-κB expression while upregulating Nrf2/HO-1 pathway activity, thereby suppressing related ferroptosis and improving SLI in mice (Zhao C. et al., 2023).

### 4.4 Alkaloids

Coptisine is a natural alkaloid with hepatoprotective effects by regulating the STAT1/IRF1/GPX4 signaling axis. Specifically, coptisine directly binds to STAT1 and inhibits its phosphorylation, thereby blocking the activation of downstream IRF1 and ultimately restoring GPX4 expression and suppressing ferroptosis, thereby alleviating SLI in mice (Zhu B. et al., 2025). Tetramethylpyrazine is an amide alkaloid extracted from Ligusticum chuanxiong. Studies have shown that tetramethylpyrazine can downregulate the expression of ferroptosis-related genes (TFR1 and Atf3), upregulate the expression of protective factors (GPX4, Nrf2, and FSP1), and mitigate SLI by inhibiting iron-phospholipid generation (Wang J. et al., 2025).

### 4.5 Saponins

Anemoside B4 is a bioactive saponin isolated from the roots of Pulsatilla chinensis. Research has indicated that anemoside B4 directly targets RAGE and inhibits the AGE/RAGE-Nrf2 axis, thereby suppressing ferroptosis and inflammation in S-ALI in mice (Bu et al., 2025).

### 4.6 Peptides/proteins

This category of substances mainly includes bioactive peptides and proteins secreted by the body itself, which can exert protective effects on sepsis-induced organ injury by regulating ferroptosis pathways. For example, irisin is a peptide generated by the hydrolysis of the FNDC5 protein, which is primarily secreted by skeletal muscle and adipose tissue. Studies have shown that irisin can effectively alleviate S-ALI in C57/BL6N mice, reducing alveolar structural damage and inflammatory responses. Its protective effects are closely related to the inhibition of ferroptosis: Irisin can reverse the oxidative stress imbalance caused by sepsis, lower ROS and Fe^2+^ levels in the lung, and downregulate the expression of ferroptosis-related markers (ACSL4, Ptgs2, and Hspa5) and proteins (ACSL4, COX-2, and p-AMPK), thereby exerting lung-protective effects (Zhang F. et al., 2025). Neuregulin 4 is a hepatoprotective lipoprotein secreted by brown adipose tissue (BAT). Research has shown that after sepsis occurs, neuregulin 4 expression increases in the BAT of mice, and plasma neuregulin 4 levels increase. Exogenous supplementation with neuregulin 4 can alleviate liver damage in septic mice by increasing the expression of GSH, SLC7A11, and GPX4 and effectively inhibiting hepatocyte ferroptosis (Feng et al., 2025).

### 4.7 Lipid mediators

Maresin 1 is a proresolving lipid mediator derived from endogenous polyunsaturated fatty acids with potent anti-inflammatory and antioxidant properties. Studies have shown that in a mouse SLI model, Maresin 1 increases the expression of Nrf2 in hepatocytes, thereby activating the downstream SLC7A11/GPX4 signaling pathway, effectively inhibiting ferroptosis and exerting hepatoprotective effects (Guo Y. et al., 2024).

### 4.8 Traditional Chinese Medicine compounds

Qishen Yiqi Pill (QSYQ) is a traditional Chinese medicinal compound composed of Astragalus, Salvia miltiorrhiza, Panax notoginseng, and Dalbergia odorifera and is known for its ability to invigorate qi and promote blood circulation. Studies have shown that QSYQ can alleviate S-ALI in mice, reduce systemic inflammatory responses, and protect the integrity of the pulmonary vascular barrier. Its protective mechanism is closely related to the inhibition of ferroptosis in pulmonary endothelial cells (Li Z. et al., 2024). Wenqingyin (WQY) is a classic traditional Chinese medicine formula used to treat inflammatory diseases. Studies have revealed that WQY can inhibit hepatocyte ferroptosis by activating the Nrf2 signaling pathway, thereby mitigating SLI (Xie et al., 2023). Xuebijing (XBJ) injection, composed of extracts from Carthamus tinctorius, *Paeonia lactiflora*, Ligusticum chuanxiong, Salvia miltiorrhiza, and Angelica sinensis, was approved in China in 2024 for the treatment of sepsis (Li C. et al., 2021). Research has shown that Xuebijing can induce ferroptosis in CCR2^hi^ monocytes by activating the Nrf2/HO-1 axis and inhibiting GPX4 expression, thereby suppressing the proinflammatory response and M1 polarization of these cells, reducing their infiltration into lungs, and ultimately alleviating sepsis-associated acute lung injury (Yang et al., 2025). Notably, the pro-ferroptotic action of XBJ in CCR2^hi^ monocytes appears to be in contrast to the general anti-ferroptotic strategy for organ protection. This discrepancy reflects cell-type specificity. In parenchymal cells (pulmonary epithelial/endothelial cells and hepatocytes), ferroptosis drives organ injury and thus needs to be inhibited. In proinflammatory monocytes/macrophages, inducing ferroptosis eliminates excessive inflammatory cells and resolves inflammation, thereby indirectly mitigating tissue damage. Such dual roles imply that ferroptosis-targeted therapy requires cell-specific precision rather than global inhibition.

### 4.9 Steroid hormones

Calcitriol is a steroid hormone produced through a two-step enzymatic hydroxylation of vitamin D in the human liver and kidneys. It plays a crucial role in maintaining bone health, regulating immunity, protecting cardiovascular function, and inhibiting tumor growth (Powała et al., 2024). Studies have shown that in an ovariectomy-induced sepsis mouse model, calcitriol exerts lung protective effects through multiple target mechanisms. On the one hand, it corrects the dysregulation of the Ang/Tie2 vascular homeostasis signaling pathway, restoring the integrity of the pulmonary vascular barrier. On the other hand, it regulates the balance of iron and copper ion metabolism, inhibiting the occurrence of ferroptosis and cuproptosis and balancing proinflammatory and anti-inflammatory responses. These mechanisms collectively significantly alleviate S-ALI in mice (Yeh et al., 2025).

Collectively, accumulated studies have verified that natural products showed therapeutic effects in S-ALI and SLI by targeting ferroptosis (Tables 2, 3). Multiple signaling pathways were involved in natural product-mediated protective effects against sepsis (Table 4).

**TABLE 4:** Mechanisms of natural products targeting ferroptosis in S-ALI and SLI.

Regulatory Mechanism/Target	Natural Products	Related Injury Model	Ref.
Keap1-Nrf2	Hyperoside	S-ALI	Chen S. et al. (2025)
	Nobiletin	SLI	Huang et al. (2023)
	Ferulic Acid	S-ALI	Tang et al. (2022)
	Andrographolide	S-ALI	Li Y. et al. (2024)
	Saikosaponin D	S-ALI	Song L. et al. (2025)
	Artemisitene	SLI	Zhao Y. et al. (2023)
	Maresin 1	SLI	Guo et al. (2024)
	XBJ	S-ALI	Yang et al. (2025)
	WQY	SLI	Xie et al. (2023)
	Anemoside B4	S-ALI	Bu et al. (2025)
NF-κB	Artemisitene	SLI	Zhao C. et al. (2023)
	Isoginkgetin	S-ALI	Lu et al. (2025)
PI3K/AKT	Wedelolactone	SLI	Yin et al. (2025)
SLC7A11/GPX4	Puerarin	S-ALI	Xu et al. (2021)
	Exocarpium Citri Grandis	SLI	Zhang G. et al. (2025)
	Wedelolactone	SLI	Yin et al. (2025)
	Rosmarinic Acid	S-ALI (ARDS)	Zeng et al. (2024)
	Curcumin	S-ALI	Chen et al. (2024)
	Tetramethylpyrazine	SLI	Wang X. et al. (2025)
	Neuregulin 4	SLI	Feng et al. (2025)
NSUN7/TFRC	Kaempferol	S-ALI	Zhang F. et al. (2025)
SIRT1	Isoferulic Acid	SLI	Gan et al. (2026)
Arachidonic Acid Metabolism	Salidroside	S-ALI	Qu et al. (2022), Zhen et al. (2024)
STAT1	Coptisine	SLI	Zhu B. et al. (2025)
ACSL4	Irisin	S-ALI	Zhang F. et al. (2025)
Ang/Tie2, Iron, and copper metabolism	Calcitriol	S-ALI	Yeh et al. (2025)

## 5 Comparative analysis of natural products and standard therapies

Although the pathophysiology mechanisms of sepsis-associated organ injuries have been well uncovered, the effectiveness of standard treatments—such as prone positioning, lung-protective mechanical ventilation, and the use of neuromuscular blocking agents—remains limited, especially in critically ill patients (Mokra et al., 2019). For SLI, treatment is largely supportive, with core interventions including infection control, initial fluid resuscitation, and hemodynamic management (Xu X. et al., 2025). Natural products targeting ferroptosis have shown great potential in the treatment of S-ALI and SLI. However, their efficacy, bioavailability, and safety need to be carefully evaluated to enhance their clinical translation value.

### 5.1 Efficacy comparison

Current therapeutic strategies against sepsis-related organ injury lack “gold standard” interventions, making it difficult to compare the efficacy of natural products in S-ALI and SLI. Recent studies have shown promising benefits of corticosteroids (CS) in clinical studies. Prolonged administration of CS has reduced the length of intensive care unit (ICU) stay, shortened the time of mechanical ventilation and hospitalization, and improved oxygenation. The underlying mechanisms of CS might be associated with their potent anti-inflammatory, antioxidant, pulmonary vasodilator, and anti-oedematous effects (Mokra et al., 2019; Annane et al., 2025).

In animal sepsis models, dexamethasone, a synthetic CS, was used as a positive control drug and showed protective effects against sepsis-induced lung and liver damage (Santos et al., 2013; He et al., 2025). Several natural products have been found to exhibit efficacy similar to dexamethasone in sepsis models at certain doses. For example, andrographolide (100 mg/kg) showed similar hepatoprotective effects with dexamethasone (1 mg/kg), as indicated by reduced ALT and AST levels. In terms of underlying mechanisms, both andrographolide and dexamethasone significantly repressed NOTCH1 and HES1 expressions. FKBP1A, a novel negative modulator of activated NOTCH1, was promoted by andrographolide. However, dexamethasone showed no significant effects on FKBP1A expression (He et al., 2025). Tangeretin is a polymethoxylated flavonoid with a rich content in citrus fruits. It exhibited significant protective effects against acute lung damage that was induced by LPS. Compared with dexamethasone, tangeretin had similar effects on reducing interstitial edema, inhibiting the infiltration of inflammatory cells, and decreasing protein content in the bronchoalveolar lavage fluid (Zhang et al., 2025a). Recently, Cao et al. compared the therapeutic effects between Reduning injection (RDN) (a Traditional Chinese Medicine formulation) and dexamethasone (5 mg/kg) in an LPS-induced rat sepsis model. Both RDN and dexamethasone suppressed serum IL-6 and TNF-α levels, promoted normalization of sepsis-induced immune dysregulation, and lowered lung, kidney, and liver pathology scores (Cao et al., 2026).

### 5.2 Bioavailability

Nowadays, natural products play important roles in rehabilitation and medicine and represent a vital source of new drugs. However, their poor solubility and low bioavailability often lead to limited absorption, insufficient distribution to target tissues, and unstable efficacy, which are major obstacles to their pharmaceutical development (Huang et al., 2024; Shi et al., 2023). For example, quercetin shows great potential in treating sepsis-related organ injury, yet its clinical application is restricted by low solubility and bioavailability resulting from its chemical structure (Wang et al., 2020; Zhou Y. et al., 2025). To overcome these limitations, researchers have developed various delivery nanosystems in recent years, such as liposomes, solid lipid nanoparticles, polymeric micelles, and extracellular vesicles (Lu Y. et al., 2023). Prodrug development or structural modification of derivatives has also been applied to selectively improve solubility and absorption (Zhou Y. et al., 2025). For example, the DQB@C nanosystem constructed by combining dihydroquercetin with hydrogen and a microalgal carrier exerts anti-inflammatory, antioxidant, and anti-ferroptotic effects as well as multi-organ protection in sepsis-associated acute lung injury, providing a novel strategy to overcome the low bioavailability of natural products and the limited clinical application of hydrogen (Wang Y. et al., 2024). These findings suggest that the pharmacokinetic disadvantages of natural products can be partially compensated by pharmaceutical technologies, yet systematic studies are still required to determine whether they can achieve clinical applicability comparable to standard therapies.

### 5.3 Safety comparison

Although antibiotics belong to the standard management of sepsis, their clinical applications are restricted by adverse effects such as nephrotoxicity (Pevzner et al., 2024). The growing rate of antibiotic resistance has also become a challenge (Yildiz et al., 2021). Glucocorticoids exert anti-inflammatory effects by inhibiting NF-κB activation and the release of broad-spectrum inflammatory factors, but their clinical application is restricted by significant safety controversies. On the one hand, glucocorticoids may increase the risk of secondary infection; on the other hand, they have even been associated with worse clinical outcomes in certain sepsis subgroups. A study in patients with sepsis-associated acute kidney injury showed that glucocorticoid treatment was significantly associated with increased ICU and in-hospital mortality (Zhu X. et al., 2025). In addition, mechanical ventilation may induce ventilator-induced lung injury, further worsening patient prognosis (Castellví-Font et al., 2024).

Natural products display distinct safety profiles in the context of sepsis. Taking XBJ injection as an example, a large-scale survey covering 93 hospitals in China and enrolling 31,913 participants showed that the incidence of adverse drug reactions (ADRs) attributed to XBJ was 0.30% (infrequent), with skin lesions being the most common, and no serious ADRs occurred, indicating favorable tolerability of XBJ (Zheng R. et al., 2019). Nevertheless, the safety of natural products should be evaluated cautiously (Fan et al., 2026). For instance, ginkgetin isomers (including isoginkgetin) carry potential hepatotoxicity and nephrotoxicity and require rigorous screening (Li Y. Y. et al., 2019).

When natural products are combined with antimicrobial agents, their interactions are bidirectional. Some natural products (e.g., capsaicin, piperine) can enhance the antibacterial efficacy of antibiotics by inhibiting bacterial efflux pumps or increasing bacterial uptake of antimicrobial agents, whereas certain combinations may weaken the efficacy of antimicrobial drugs, leading to treatment failure or the need for higher doses (Malczak and Gajda, 2023). This suggests that when natural products are used as adjuvant therapy for sepsis, their interactions with antibiotics should be systematically evaluated to ensure the safety and efficacy of combination therapy.

## 6 Safety of natural products in preclinical and clinical settings

Toxicological evaluation is one of the key processes in modern drug research. The toxic effects of certain natural products have been identified (Table 5).

**TABLE 5:** Toxic effects of ferroptosis-targeting natural products in sepsis-associated organ injury.

Natural products	Source	Toxic target organ/System	Toxic effects	Experimental model	Dose/Concentration	Ref.
Andrographolide	Paniculata	Lung, Liver, Kidney	Induced alveolar structure destruction, renal tubular edema, and liver damage	Mice	0–100 mg/kg with repeated administration	Lu et al. (2026)
		Kidney	Inhibited proliferation of human renal tubular epithelial cells, induced apoptosis, oxidative stress and endoplasmic reticulum stress	Human Renal Tubular Epithelial Cells (HK-2)	0–250 μmol/L	Gu et al. (2016)
		Kidney	induced lumbar pain, decreased urine output, nausea, vomiting, and caused pathological manifestations of acute tubular necrosis	Patients with acute kidney injury	100–750 mg (58% were 500 mg) by intravenous drip once a day	Zhang et al. (2014)
		Reproductive System	Disrupted spindle organization and migration, actin cap formation and cytokinesis; induced oocyte apoptosis; reduced fertilization rate; interfered with cytoskeletal rearrangement and meiotic maturation	Female Rodent Oocytes	20 μM	Liang et al. (2017)
Saikosaponin D	Bupleuri Radix	Liver	Inhibited hepatocyte viability, induced apoptosis	LO2 Human Hepatocytes, Mice	*In vitro*: 0.4–2 μM; *In vivo*: 300 mg/kg, oral administration, for 1 week	Chen et al. (2013), Zhang et al. (2016)
		Nervous System	Decreased cognitive function, inhibited hippocampal neurogenesis; inhibited proliferation and survival of neural stem/progenitor cells; induced neuronal apoptosis	Primary Neural Stem/Progenitor Cells, Neonatal Cortical Neurons, Mice	*In vivo*: intragastric administration, 4 and 8 mg/kg (7 days), 16 mg/kg (14 days); *In vitro*: 2–4 μM, 1.28–19.2 μM	Lixing et al. (2018), Qin et al. (2019), Qin et al. (2020), Zheng et al. (2019)
		Hematological System	Concentration-dependent hemolysis	Human Red Blood Cells, Sheep Red Blood Cells	Human red blood cells: 0.64–1.92 μM; Sheep red blood cells: hemolysis began at ≥1.28 μM	Abe et al. (1978), Nose et al. (1989)
Isoginkgetin	Ginkgo Biloba Leaves	Liver, Kidney	induced hepatocellular hydropic degeneration, significantly increased alkaline phosphatase activity; renal tubular, glomerular, and interstitial damage	Human Renal Tubular Epithelial Cells (HK-2), Normal Hepatocytes (L-02) and Mice	20 mg/kg/day	Li X. et al. (2019)
Coptisine	Coptis Chinensis	Liver, Kidney, Lung	caused cytotoxicity on HepG2 and 3T3-L1 cells, significant weight loss in female rats, hepatocellular steatosis, and pulmonary inflammatory cell infiltration	HepG2, 3T3-L1 Cells; Mice (Acute Toxicity), Rats (90-day Subchronic Toxicity)	*In vitro*: 1–160 μg/mL Acute: 579, 694, 833, 1,000, 1,200 mg/kg; Subchronic: 156 mg/kg/day for 90 consecutive days	Yi et al. (2013)
Calcitriol	Active Metabolite of Vitamin D	Calcium Metabolism System (Hypercalcemia)	Self-limiting Grade 1 hypercalcemia	Phase I Clinical Trial: Patients with Refractory Malignant Tumors (n = 15)	Starting dose: 0.06 μg/kg/week Maximum tested dose: 2.8 μg/kg/week	Beer et al. (2001)
		Systemic Toxicity, Kidney	90% mortality rate; weight loss; lethargy; mild bilateral renal tubular calcification	Athymic Nude Mice Xenografted with Y-79 Human Retinoblastoma	0.05 μg/mouse/time, intraperitoneal injection, 5 times a week for 5 consecutive weeks	Sabet et al. (1999)
Hyperoside	Perforatum L	Kidney (Long-term High Dose)	Low acute toxicity (LD50 > 5,000 mg/kg), slowed down fetal mouse growth; caused reversible nephrotoxicity with long-term use	Mice/Rats	No specific dose specified	Ai et al. (2012), Xu et al. (2022)

For example, andrographolide exhibits multi-organ toxicity in multiple studies. In rodents, repeated administration of andrographolide (≤100 mg/kg) induces liver and kidney injuries, manifested as elevated ALT and AST levels and renal tubular edema. Notably, no acute toxicity was observed after a single administration at this dose, whereas toxicity occurred following repeated dosing (Lu et al., 2026). *In vitro* experiments showed that andrographolide inhibited the proliferation of human renal tubular epithelial cells and induced apoptosis through mediating oxidative stress and endoplasmic reticulum stress pathways (Gu et al., 2016). In clinical practice, intravenous administration of andrographolide has been associated with acute tubular necrosis, with 58% cases with kidney injury occurring at a dose of 500 mg/d (Zhang et al., 2014). Furthermore, it affects the reproductive system by interfering with oocyte meiosis and reducing fertilization rates (Liang et al., 2017).

Saikosaponin D exerts well-defined toxic effects across multiple systems. Hepatotoxicity can be induced by saikosaponin D both *in vitro* (IC_50_ = 2.14 μM) and in mice (300 mg/kg), evidenced by enhanced apoptosis via mitochondrial and death receptor pathways (Chen et al., 2013; Zhang et al., 2016). In the nervous system, oral administration of saikosaponin D to mice at 4 and 8 mg/kg for 7 days or 16 mg/kg for 14 days leads to cognitive decline, suppressed hippocampal neurogenesis, and inhibited proliferation and survival of neural stem/progenitor cells. *In vitro*, saikosaponin D at concentrations of 2–4 μM can induce neuronal apoptosis (EC_50_ = 2.92 μM) (Lixing et al., 2018; Qin et al., 2019; Qin et al., 2020; Zheng J. et al., 2019). In the hematological system, saikosaponin D exerts concentration-dependent hemolysis, occurring in human erythrocytes at 0.64–1.92 μM and in ovine erythrocytes at ≥1.28 μM (Abe et al., 1978; Nose et al., 1989).

Puerarin has been associated with 129 reported adverse reactions in clinical applications, including 13 deaths, involving the immune, digestive, cardiovascular, and urinary systems. The most adverse reactions (88.3%) were associated with the immune and circulatory systems. In addition, 61.1% side effects occurred at 48 h after puerarin administration. However, the severity of adverse reactions is not dependent on the dosage of puerarin but is associated with the solvent (Liu et al., 2023).

Coptisine has an LD_50_ of 852.12 mg/kg in mouse acute toxicity tests, indicating mild toxicity and relative safety at conventional doses. However, acute toxicity may occur at high exposure levels near or exceeding the LD_50_. In a 90-day subchronic toxicity study, rats administered with coptisine (156 mg/kg/day in feed) exhibited body weight loss, altered liver and kidney function parameters, and histopathological changes in the liver, indicating that toxicity manifests only under long-term, continuous exposure rather than single dosing. At the cellular level, coptisine exerts dose-dependent cytotoxicity in HepG2 and 3T3-L1 cells, with IC_50_ values of 64.81 μg/mL and 56.48 μg/mL, respectively, showing cytotoxicity only at relatively high concentrations (Yi et al., 2013).

Calcitriol exhibits marked dose- and species-dependent toxicity. In a Phase I clinical trial in patients with refractory malignant tumors (n = 15), the drug only induced self-limiting Grade 1 hypercalcemia with mild and reversible hypercalcemia, with no Grade 3 or higher toxicities, demonstrating clinically acceptable safety (Beer et al., 2001). However, in a Y-79 human retinoblastoma xenograft nude mouse model (0.05 μg per mouse, intraperitoneal injection, 5 times weekly for 5 weeks), the drug caused 90% mortality, weight loss, lethargy, and mild bilateral renal tubular calcification, suggesting significantly increased systemic and nephrotoxicity risks under specific high-dose or long-term administration conditions (Sabet et al., 1999).

Hyperoside demonstrates favorable overall safety, with an acute toxicity LD_50_ > 5,000 mg/kg in mice and no genotoxicity (evidenced by a negative Ames test). Its toxicity mainly appears under specific conditions: long-term high-dose use may cause reversible nephrotoxicity, and gestational administration, although having little effect on pregnant mice, can slow fetal growth. This indicates that the compound is safe for single use at conventional doses, but nephrotoxicity risks with long-term use and effects on embryonic development during pregnancy require attention (Ai et al., 2012; Xu et al., 2022).

Kaempferol did not induce significant changes in body weight, organ weight, hematology, liver and kidney function, cardiac-related biochemical parameters, or oxidative stress markers in mice after 28 days of oral administration (3, 10, 30 mg/kg), with no histopathological abnormalities observed in the liver, kidney, heart, or lung (Yangzom et al., 2022). Nobiletin showed no obvious cytotoxicity *in vivo* (0.05% dietary concentration for 3–20 weeks) or *in vitro* (effective concentration 40 μM), and cells resumed normal growth after withdrawal, with safety superior to existing chemotherapeutic agents (Goh et al., 2019). Rosmarinic acid did not cause abnormalities in blood cells or liver/kidney function in healthy volunteers at single doses of 100–500 mg (Noguchi-Shinohara et al., 2015). Curcumin showed no obvious toxicity in humans at 6 g/day for 4–7 weeks or 500 mg twice daily for 30 days, with only mild gastrointestinal discomfort reported in a few individuals (Soleimani et al., 2018). Its nanoformulations exhibited no carrier toxicity in normal cells (MCF 10A) and selective toxicity in 4T1 cancer cells (Gholibegloo et al., 2019). Anemoside B4 did not induce changes in body weight, AST, ALT, creatinine, or urea nitrogen levels in mice (2.5 g/kg, intraperitoneal injection for 14 days), and showed no effects on the viability of human embryonic kidney 293 cells *in vitro* (He et al., 2019). Salidroside showed no obvious toxicity in rats after repeated administration for 7 and 28 days (500, 1,000, 2000 mg/kg) and no clinical adverse events in humans at 600 mg/day (Kasprzyk et al., 2022; Zhong et al., 2018; Zhang et al., 2012).

## 7 Barriers of natural products in clinical application

### 7.1 Poor solubility and low bioavailability

Poor solubility and low bioavailability represent the most prevalent barriers to the clinical application of natural products across diverse structural classes. As a monomeric flavonoid, quercetin exerts multiple pharmacological effects, including regenerative, anti-inflammatory, and immunomodulatory activities, yet its poor water solubility and low bioavailability severely limit clinical translation (Yang et al., 2024). Similarly, the isoflavonoid glycoside puerarin faces translational challenges due to insufficient water solubility and bioavailability (Wu et al., 2018; Zhao Y. et al., 2023). Kaempferol is typically ingested as highly polar glycosides, resulting in extremely low oral bioavailability (approximately 2%) (Yao et al., 2024). Nobiletin and wedelolactone are also limited by low solubility and bioavailability (Ning et al., 2023; Sun L. et al., 2023). Notably, as a lipophilic polymethoxylated flavonoid, nobiletin is further restricted by poor stability and pH sensitivity in addition to low solubility (Li H. et al., 2025).

Among phenolic acids, ferulic acid suffers from poor water solubility and limited bioavailability, while rosmarinic acid shows low gastrointestinal absorption (only 6.3% within 48 h) and is prone to metabolism by intestinal microbial esterases (Purushothaman and Rizwanullah, 2024; Calabrese et al., 2024). Furthermore, curcumin exhibits extremely poor water solubility at room temperature, a half-life of less than 10 min at neutral pH (7.2), and disadvantages including poor oral absorption and short plasma half-life, leading to extremely low bioavailability (Kotha and Luthria, 2019; Hao et al., 2023). Andrographolide and saikosaponin D also show low oral absorption due to poor water solubility, hindering the development of effective oral formulations (Yen et al., 2020; Zhou et al., 2021). The artemisinin derivative artemisitene has an oral absolute bioavailability of only 3.7% in rats, indicating poor oral absorption and significant first-pass metabolism, representing a major limitation for its clinical development (Wu et al., 2017). Tetramethylpyrazine also faces challenges of poor water solubility and low bioavailability (Jia et al., 2025). In addition, the pentacyclic triterpenoid saponin anemoside B4, despite potent anti-inflammatory and antioxidant activities, exhibits low oral bioavailability that limits its application potential (Wen et al., 2026).

### 7.2 Metabolic instability and rapid clearance *in vivo*

Many natural products undergo rapid metabolism or degradation *in vivo*, resulting in short effective exposure durations. For example, ferulic acid is unstable and rapidly metabolized under physiological conditions (Purushothaman and Rizwanullah, 2024); 75% of rosmarinic acid is excreted in urine within 6 h of administration, limiting its effective exposure (Calabrese et al., 2024). Curcumin is rapidly metabolized *in vivo* mainly via reduction, glucuronidation, and sulfation (Kotha and Luthria, 2019). Coptisine undergoes extensive multi-pathway metabolism *in vitro* and *in vivo*, with a short half-life in rats (T_1/2_ = 0.71 h) (Su et al., 2015). Moreover, curcumin is prone to solvolysis, auto-oxidation, and photodegradation, requiring strict storage conditions (Kotha and Luthria, 2019), while rosmarinic acid is easily metabolized by intestinal microbial esterases (Calabrese et al., 2024).

### 7.3 Absorption and distribution barriers

Specific tissue barriers (e.g., the blood–brain barrier) and poor intestinal absorption represent key limitations to therapeutic efficacy. As a natural antioxidant, the neuroprotective effects of ferulic acid are restricted by poor blood–brain barrier permeability (Puris et al., 2019). Rosmarinic acid cannot penetrate the blood–brain barrier via oral administration, and even after intraperitoneal or intravenous injection, only extremely low levels are detected in the brain, failing to reach effective pharmacological concentrations (Calabrese et al., 2024). Tetramethylpyrazine is also limited by poor blood–brain barrier permeability in central nervous system disorders (Puris et al., 2019). Regarding intestinal absorption, pharmacokinetic studies of wedelolactone indicate poor intestinal absorption (Upadhyay et al., 2012). Coptisine shows low gastrointestinal absorption, with most excreted unchanged in feces (Lu Q. et al., 2023).

### 7.4 Toxic and side effects associated with specific administration routes

For injectable formulations, safety issues represent major clinical obstacles. Intravascular administration of puerarin may induce various adverse reactions, among which hemolysis is a key factor limiting its clinical application, along with drug fever, rash, liver and kidney injury, anaphylactic shock, etc. (Zhao Y. et al., 2023). Meanwhile, puerarin possesses estrogenic properties, and long-term use may increase breast cancer risk, raising concerns about long-term endocrine effects (Ou et al., 2025). In addition, coptisine exhibits potential hepatotoxicity, representing a major safety concern for its clinical application (Lu Q. et al., 2023). The clinical use of calcitriol is mainly limited by its severe side effect of hypercalcemia (Pełczyńska et al., 2005).

### 7.5 Challenges in source, production, and formulation

Some natural products are difficult to achieve clinical translation and widespread application due to scarce natural sources, challenging synthesis, or poor product consistency. The natural production of SAL is limited by plant growth conditions, low content, and difficult extraction, while chemical synthesis is hindered by its complex structure, lengthy procedures, and high cost, leading to supply–demand imbalance (Sun et al., 2025). Furthermore, Chinese herbal compound preparations face clinical application difficulties due to complex components, difficulty ensuring consistent product quality, and risks of potential herb–herb interactions and adverse reactions (Ji et al., 2025).

## 8 Summary and prospects

This review comprehensively discusses the central role of ferroptosis in S-ALI and SLI, along with its molecular mechanisms, and highlights the latest research progress on natural products targeting ferroptosis in the treatment of S-ALI and SLI. Substantial evidence indicates that during sepsis, typical ferroptosis characteristics occur in the lung and the liver. By regulating key targets in the ferroptosis process, natural products effectively reduce Fe^2+^ accumulation and the collapse of the antioxidant defense system, lipid peroxidation, and reactive oxygen species (ROS), ultimately mitigating damage to pulmonary microvascular endothelial cells, alveolar epithelial cells, and hepatocytes.

Natural products, leveraging their multitarget advantages, can exert multidimensional regulatory effects on complex pathological processes, demonstrating significant therapeutic potential. As discussed in this article, various natural active compounds, such as flavonoids, saponins, and phenolic acids, can effectively inhibit ferroptosis in organ tissues through key mechanisms, including activation of the Nrf2 signaling pathway, suppression of ACSL4 activity, modulation of the autophagy–ferroptosis axis, direct scavenging of ROS, and regulation of iron homeostasis. Importantly, many natural products can synergistically exert anti-inflammatory and antioxidant effects, disrupting the vicious cycle of sepsis-induced organ damage at multiple stages. This provides a rich pool of candidate molecules and a solid theoretical foundation for the development of novel therapeutic agents.

Future research in this field should focus on the following directions. First, at the level of mechanism exploration, most current studies concentrate on classical pathways such as the Nrf2/GPX4 pathway. However, the regulatory network of ferroptosis is far more complex than is currently known. Notably, a series of emerging mechanisms, such as the VDR/FFAR2 (Xu et al., 2024) axis and HIF-1α/LDHA axis (Kuang et al., 2025), have been confirmed as key pathways regulating cellular ferroptosis sensitivity. However, how natural products act on these emerging targets and their pharmacological mechanisms remain unclear. To accelerate the discovery of potential targets, artificial intelligence (AI)-driven drug screening platforms hold great promise. AI-based virtual screening, molecular docking, and machine learning models can efficiently predict potential interactions between natural products and ferroptosis-related targets, thereby enabling high-throughput prioritization of candidate compounds for further experimental validation. Second, most existing research remains at the stage of cell and animal models. Although encouraging findings have been observed in these models, there are significant physiological and pathological differences between cell/animal models and humans. For example, the pathogenesis of sepsis-induced organ injury involves complex factors such as genetics, which are difficult to fully replicate in current models. Therefore, there is an urgent need to validate their efficacy in animal models that more closely resemble human diseases (e.g., aged animals or models with comorbid conditions) and to promote high-quality multicenter clinical trials to assess their safety and efficacy in sepsis patients.

Additionally, defining the “damage threshold” of ferroptosis is a core challenge for achieving precise intervention. The initiation of ferroptosis requires a critical damage threshold, which is determined by the dynamic balance of multiple factors, such as the intracellular free iron concentration, lipid peroxidation levels, GSH reserves, and GPX4 activity. Future studies need to quantify this threshold. For instance, determining during different stages of sepsis at what level of GSH in hepatocytes or alveolar epithelial cells must decrease or to what concentration of lipid peroxides must accumulate before ferroptosis is irreversibly triggered. Finally, at the therapeutic strategy level, combination drug regimens should be actively explored. By clarifying the damage threshold and signaling networks, exploring the combined application of natural products with existing antibiotics, anti-inflammatory drugs, or other cell death inhibitors (e.g., apoptosis or pyroptosis inhibitors) may yield synergistic effects, providing superior comprehensive treatment options for multiorgan protection in sepsis. In addition, the development of targeted delivery systems (such as nanoparticle-based formulations) represents a research direction of critical translational value. Given the poor water solubility, low bioavailability, and off-target effects of many natural products, advanced drug delivery platforms—including liposomes, polymeric nanoparticles, and biomimetic nanocarriers—can significantly improve their stability, enhance targeting efficiency toward inflamed lung and liver, and enable controlled release properties. This maximizes therapeutic efficacy while minimizing systemic toxicity.

In summary, natural products targeting ferroptosis have opened up promising new avenues for the prevention and treatment of sepsis-related organ injury. Through in-depth mechanistic research, particularly the quantitative understanding of the ferroptosis trigger threshold, and overcoming translational bottlenecks via AI-assisted drug discovery and advanced therapeutic nanodelivery systems, these natural products are expected to become important components of precision therapeutic strategies for sepsis in the future.
